# Safety Testing of an Antisense Oligonucleotide Intended for Pediatric Indications in the Juvenile Göttingen Minipig, including an Evaluation of the Ontogeny of Key Nucleases

**DOI:** 10.3390/pharmaceutics13091442

**Published:** 2021-09-10

**Authors:** Allan Valenzuela, Claire Tardiveau, Miriam Ayuso, Laura Buyssens, Chloe Bars, Chris Van Ginneken, Pierluigi Fant, Isabelle Leconte, Annamaria Braendli-Baiocco, Neil Parrott, Georg Schmitt, Yann Tessier, Paul Barrow, Steven Van Cruchten

**Affiliations:** 1 Comparative Perinatal Development, Department of Veterinary Sciences, University of Antwerp, 2610 Wilrijk, Belgium; allan.valenzuela@uantwerpen.be (A.V.); miriam.ayusohernando@uantwerpen.be (M.A.); laura.buyssens@uantwerpen.be (L.B.); chloe.bars@uantwerpen.be (C.B.); chris.vanginneken@uantwerpen.be (C.V.G.); 2 Charles River Laboratories France Safety Assessment SAS, 69210 Saint-Germain-Nuelles, France; tardiveau.claire@gmail.com (C.T.); pierluigi.fant@crl.com (P.F.); isabelle.leconte@crl.com (I.L.); 3 Roche Pharmaceutical Research and Early Development, F. Hoffmann-La-Roche, Ltd., 4070 Basel, Switzerland; annamaria.braendli-baiocco@roche.com (A.B.-B.); neil_john.parrott@roche.com (N.P.); georg.schmitt@roche.com (G.S.); yann.tessier@roche.com (Y.T.); paul.barrow.pb1@roche.com (P.B.)

**Keywords:** antisense oligonucleotide (ASO), endonuclease, exonuclease, locked nucleic acid (LNA), Göttingen Minipig, pediatric safety assessment, RNase H

## Abstract

The adult Göttingen Minipig is an acknowledged model for safety assessment of antisense oligonucleotide (ASO) drugs developed for adult indications. To assess whether the juvenile Göttingen Minipig is also a suitable nonclinical model for pediatric safety assessment of ASOs, we performed an 8-week repeat-dose toxicity study in different age groups of minipigs ranging from 1 to 50 days of age. The animals received a weekly dose of a phosphorothioated locked-nucleic-acid-based ASO that was assessed previously for toxicity in adult minipigs. The endpoints included toxicokinetic parameters, in-life monitoring, clinical pathology, and histopathology. Additionally, the ontogeny of key nucleases involved in ASO metabolism and pharmacologic activity was investigated using quantitative polymerase chain reaction and nuclease activity assays. Similar clinical chemistry and toxicity findings were observed; however, differences in plasma and tissue exposures as well as pharmacologic activity were seen in the juvenile minipigs when compared with the adult data. The ontogeny study revealed a differential nuclease expression and activity, which could affect the metabolic pathway and pharmacologic effect of ASOs in different tissues and age groups. These data indicate that the juvenile Göttingen Minipig is a promising nonclinical model for safety assessment of ASOs intended to treat disease in the human pediatric population.

## 1. Introduction

Antisense oligonucleotides (ASOs) belong to a therapeutic modality designed to treat specific diseases by selectively modulating the gene expression of disease-associated proteins. Usually 12–24 nucleotides in length, ASOs are designed to hybridize with a specific and complementary mRNA, resulting in inhibition of protein translation [1]. Currently, more than a dozen RNA-targeting therapeutics are authorized for use, while many others are in development for various indications for which no or limited treatment options are available [2,3,4].

One mechanism by which ASOs inhibit RNA translation is through RNA degradation by the RNase H-dependent cleavage mechanism [5,6]. RNase H-dependent ASOs utilize a ubiquitous endogenous ribonuclease that specifically hydrolyzes the RNA strand in the RNA–DNA heteroduplexes. This antisense mechanism remains one of the most utilized despite the rapid advancements in RNA-targeted therapeutics [7,8]. After parenteral administration, ASOs transiently bind to plasma proteins before getting biodistributed rapidly to peripheral tissues. The plasma protein interaction supports tissue bioavailability and reduces renal clearance [9,10]. As observed across several mammalian species, the highest tissue concentration is reached primarily in the kidney and liver, with a long elimination phase from those tissues [11,12]. Accumulation-related changes in these organs are commonly observed in nonclinical testing of ASOs together with hematological alterations, immunostimulation, and coagulopathy [13].

Unlike conventional small molecule drugs, ASOs do not appear to be a substrate for cytochrome P450 enzymes [14,15]. Instead, ASOs are hydrolyzed by endogenous nucleases in the blood and other tissue compartments [16,17]. Nucleases are phosphodiesterases that cleave the phosphodiester bonds (P-O) of nucleic acids and can be classified as either exonucleases, which cleave one nucleotide at the 3′- or 5′-end; or endonucleases, which cleave P-O in the middle of the nucleic acid chain [18]. ASOs are metabolized by 3′-exonucleases while in circulation [19,20,21]. Once ASOs reach the various tissue compartments, they are generally metabolized by endonucleases followed by exonucleases [21]. Unmodified ASOs containing P-O bonds are inherently susceptible to nucleolytic degradation and have poor intrinsic binding affinity and biodistribution, which make these ASOs not suitable as therapeutic agents. This led to the development of first-generation ASOs with backbone chemistry modifications, e.g., phosphorothioate (PS), so-called phosphorothioate antisense oligonucleotides (PTOs) [22,23]. PTOs later included additional modifications of the nucleotide sugar moiety, e.g., locked-nucleic acids (LNAs) in the flanking regions. This modification pattern, termed as gapmers, results in greater nuclease resistance and improved pharmacokinetics and potency [24] while still ensuring the activation of RNase H [25,26,27]. Currently, additional modifications are being investigated to further improve intracellular uptake of ASOs and delivery to the target tissues by e.g., covalently binding them to a carrier or ligand [23,28,29].

To date, no specific guidelines regulate the nonclinical safety testing of ASO drug candidates. The non-clinical testing guidelines for small molecule drugs are applicable since ASOs are manufactured by chemical synthesis. Thus, repeat-dose toxicity studies in both a rodent and a non-rodent are generally required [30], for which one needs to be pharmacologically relevant. Classically, non-human primates (NHPs) are the preferred non-rodent model for this class of compounds when the candidate ASO does not hybridize in dogs. However, the adult Göttingen Minipig appears to be a suitable alternative to NHPs, as it showed a similar safety profile in a previous study [31]. Moreover, with the sequencing of the Göttingen Minipig genome [32,33], it has become feasible to synthesize homologous ASOs that cross-react in swine to allow the evaluation of adverse effects related to the pharmacological target (exaggerated pharmacology). In essence, pharmacological target homology is crucial for demonstrating pharmacological effects of ASO candidates in the relevant nonclinical species. Still, only 14% of RNase-H-based ASOs and 20–40% of other ASO subclasses were tested in minipigs for non-rodent toxicity studies. ASOs are also of interest for pediatric indications, including neuromuscular diseases [34,35,36], such as spinal muscular atrophy [37] and Duchenne muscular dystrophy [38], which have already been treated successfully [6]. Moreover, ASOs are also being explored for the treatment of several other diseases in children such as retinopathy of prematurity [39], leukodystrophies [40], and inherited lung diseases [41]. Consequently, more repeat-dose toxicity studies in juvenile animals may be required/expected before starting pediatric clinical trials, depending on the intended pediatric age group(s), duration of treatment, etc. [42]. This is motivated by concerns of increased susceptibility of juveniles to toxicities due to, for instance, differences in absorption, distribution, metabolism, and excretion (ADME) [43]. Generally, minipigs and humans share many developmental milestones [42]. Moreover, both species show comparable differences between adult and juvenile populations. Therefore, the juvenile minipig as a model species presents multiple advantages in view of developmental pharmacology, drug discovery, and drug safety testing [44]. For ASOs, the juvenile minipig is a promising model due to its biological similarity to humans, technical feasibility, welfare considerations, and rapid postnatal development [45,46]. However, to date, there are no data available on the pharmacodynamic, toxicological, and metabolic profiles of ASOs in the juvenile minipig.

To qualify the use of the juvenile Göttingen Minipig for pediatric safety assessment of ASOs, knowledge of the ontogeny of the key nucleases responsible for ASO metabolism and pharmacologic activity is pivotal. The functional immaturity of nucleases for ASO metabolism may result in high ASO exposure, resulting in more severe or additional toxicities in young individuals. On the other hand, the functional immaturity of RNase H may lead to low or absent ASO pharmacologic activity or could mask potential exaggerated pharmacology or off-target effects. Juvenile animal studies are designed to detect such effects and thus provide valuable safety data for the human pediatric population.

The goal of this study was to assess potential differences in exposure/toxicity and pharmacologic effect (i.e., reduce total and LDL cholesterol) of a model ASO in the juvenile Göttingen Minipig, following weekly dosing starting on postnatal day (PND) 1 for up to 8 weeks. This was done using an antisense LNA/PS/LNA gapmer (RTR5001) that had been previously characterized in adult Göttingen Minipigs and NHPs, in which the kidney and the liver were the primary organs of distribution/toxicity [31,47]. The ontogeny of nuclease expression and activity was assessed in blood, kidney, and liver tissues obtained from the study animals and in additional tissues from a biobank (gestational day (GD) 84–86, 108; PND 1, 3, 7, 28; and adults). Quantitative polymerase chain reaction (qPCR) and activity assays were performed using three isosequential ASOs (i.e., unmodified, all-PS, LNA/PS/LNA gapmer) to explore the findings of the in vivo study with regard to the toxicokinetic and pharmacologic effects of ASOs.

## 2. Materials and Methods

### 2.1. Antisense Oligonucleotides

RNase H-active antisense LNA/PS/LNA gapmer (RTR5001) with the 14-base pair sequence 5′-TGCtacaaaacCCA-3′ (upper-case, LNA monomers; lower-case, PS DNA monomers) was provided by Roche Innovation Center (Copenhagen, DK). RTR5001 targets the proprotein convertase subtilisin/kexin 9 (*PCSK9*) mRNA (NCBI reference sequence: NM_174936.3) that is mainly expressed in the liver (therapeutic target organ) [48]. This model ASO matches the human sequence while having a single end-standing mismatch to the minipig sequence, which does not ablate its pharmacology in swine [31]. Two additional isosequential variants (unmodified and all-PS modified) in desalted form were procured from IDT (Leuven, BE).

### 2.2. Study Design

**Figure 1:**
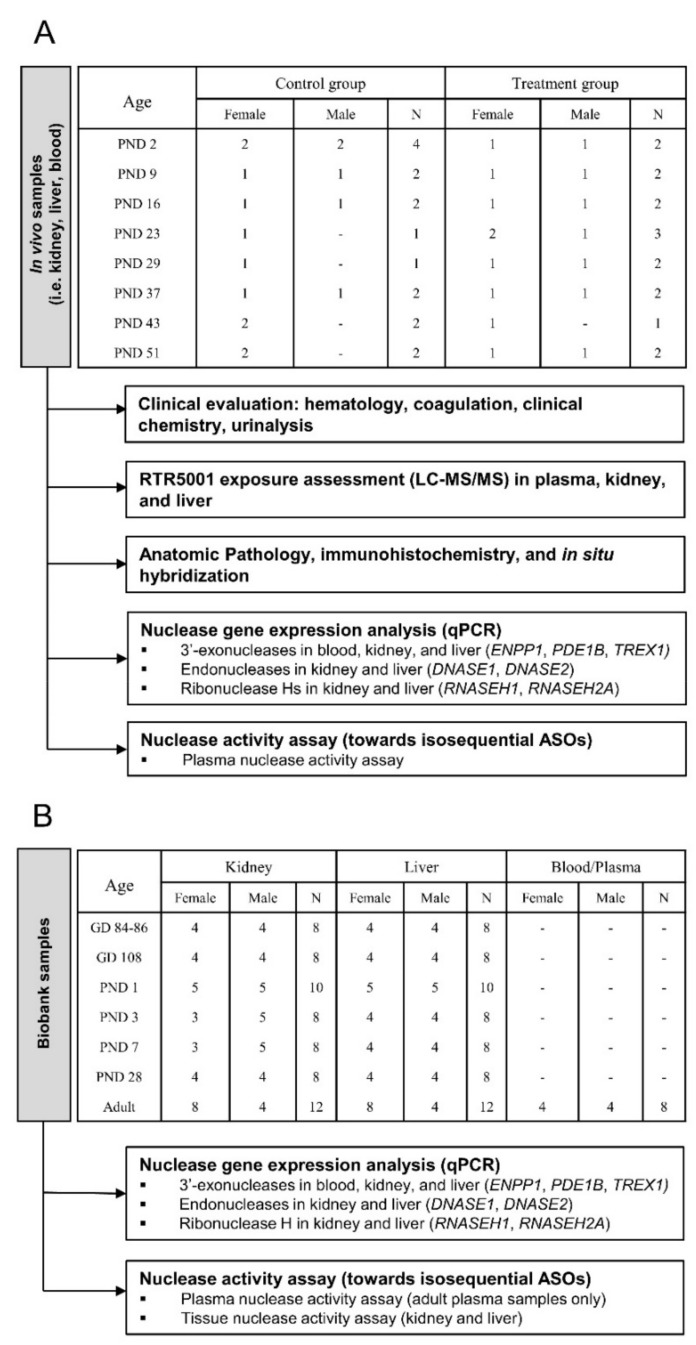
Schematic diagram of the study design illustrating the samples used and main experimental approaches. (**A**) In vivo study sample distribution over the different developmental stages, experimental groups, and sex. (**B**) Biobank study sample distribution over different juvenile and adult stages, and sex. Downstream usage of samples for the different assays are represented by arrows. GD: gestational day; PND: postnatal day; qPCR: quantitative polymerase chain reaction; *ENPP1*: ectonucleotide pyrophosphatase/phosphodiesterase 1; *PDE1B*: Phosphodiesterase 1B; *TREX1*: three-prime repair exonuclease 1; *DNASE1* and *DNASE2*: deoxyribonuclease 1 and 2; *RNASEH1* and *RNASEH2A*: ribonuclease H 1 and 2 subunit A; LC-MS/MS: liquid chromatography coupled with tandem mass spectrometry.

Two experiments were set up: (1) an in vivo 8-week repeat-dose toxicity study in juvenile Göttingen Minipigs (from now on referred to as the in vivo study; Figure 1A); and (2) an investigation of the ontogeny of nuclease gene expression and activity in the blood, kidney, and liver of different juvenile and adult Göttingen Minipigs (from now on referred to as the biobank study; Figure 1B). In-life monitoring, toxicokinetic parameters, clinical and anatomic pathology (including immunohistochemistry and in situ hybridization) after model ASO (RTR5001) administration were investigated in the in vivo study. Then, the ontogeny of nucleases was evaluated in blood and tissue samples from the in vivo study together with additional samples from our biobank.

### 2.3. In Vivo Study

The in vivo repeat-dose toxicity study of RTR5001 in animals was carried out at Charles River Laboratories France Safety Assessment SAS. The test facility is accredited by AAALAC, and the study was conducted according to standard operating procedures in accordance with OECD Good Laboratory Practice (GLP). The welfare and treatment of animals were in accordance with the following: Guide for the care and use of laboratory animals, 2011; Decree no. 2013–118 relating to the protection of animals used in scientific experiments described in the Journal Officiel de la République Française on 1 February 2013; and Directive 2010/63/EU of the European Parliament and of the Council of 22 September 2010 on the protection of animals used for scientific purposes.

Four multiparous pregnant Göttingen Minipig sows aged between 21 and 32 months old were supplied by Ellegaard Göttingen Minipigs A/S (Dalmose, Denmark). The sows were acclimatized to the study housing conditions for three weeks before the predicted parturition date. After birth, the piglets were given 1 mL of iron intramuscularly within 24 h and were allowed to suckle the dam until weaning. Sows were identified by ear tags and the piglets by transponder implants. Each sow and her litter were housed separately in 4 m^2^ pens with anti-crush protection until PND 28 (weaning). After weaning, the litters were grouped by sex in two 6 m^2^ pens.

Thirty-two piglets from the four litters were randomly allocated to a control and an RTR5001 treatment group. RTR5001 was administered subcutaneously caudal to the pinna (left and right alternated) at a dose of 20 mg/kg and a volume of 2.5 mL/kg on PND 1, followed by seven weekly doses (i.e., PND 8, 15, 22, 28, 36, 42, and 50). Control animals received the same volume of vehicle (sterile 0.9% NaCl). A homogenous distribution of the litters across the different groups was ensured by randomly allocating piglets from the same litter to the different studied time points (i.e., 24 h after each dosing: PND 2, 9, 16, 23, 29, 37, 43, and 51) (Figure 1A). Control and treated animals were humanely killed at their designated time points by intravenous injection of sodium pentobarbital followed by exsanguination.

#### 2.3.1. In-Life Monitoring

The following parameters and endpoints were closely monitored throughout the study period: mortality, clinical observations, body weight development, food consumption, and physical development. Body weight was measured daily from PND 1 to 7 and then twice per week until the end of the study period. All piglets were checked for landmarks of physical development (i.e., pinna unfolding, incisor eruption, and eye opening) from birth until PND 1–2, by which point all piglets had attained all three milestones.

#### 2.3.2. Tissue Sampling

Liver and kidney samples for bioanalytical examination, gene expression, and nuclease activity assays were harvested, weighed, and immediately snap-frozen in liquid nitrogen before storage at −80 °C. Blood samples were collected from the unfasted piglets during necropsy from the external jugular vein. Blood samples for the gene expression experiment were collected with EDTA-K_2_ as anticoagulant and centrifuged (1000× *g*) for 15 min at 4 °C. The sediments (buffy coat and RBC) were mixed with 1 mL lysis buffer DL (Nucleospin^®^ RNA Blood Midi). Blood samples for the nuclease activity assay were collected with sodium citrate as an anticoagulant and centrifuged (15,000× *g*) for 5 min at 4 °C. The plasma samples were aliquoted and stored at −80 °C. Blood was collected with EDTA-K_2_ as anticoagulant and centrifuged (1800× *g*) for 10 min at 4 °C for plasma exposure assessment at several time points (0, 1, 3, 6, 24 h) after each dosing and throughout the study.

#### 2.3.3. Exposure Assessment

Plasma, liver, and kidney cortex tissue were analyzed by liquid chromatography coupled to tandem mass spectrometry (LC-MS/MS) using an LC Shimadzu system coupled with an API 6500+ Mass Spectrometer (AB Sciex, Framingham, MA, USA). Before analysis, tissue samples were homogenized with water (dilution factor 4), and the aliquot homogenates were diluted 5-fold in minipig blank plasma before sample preparation. The RTR5001 quantification was performed against a minipig plasma calibration curve from 1.00 to 1000 nM. The performance of sample analysis was monitored by analyzing quality control samples in minipig plasma spiked with a known concentration (2.00, 50.0, and 750 nM) of RTR5001. Fifty µL of calibration standards, quality control samples (freshly prepared in minipig plasma), and tissue homogenate samples diluted in minipig blank plasma were treated for protein denaturation with 150 µL of 4 M guanidine thiocyanate after the addition of 10 µL of the internal standard (1000 ng/mL or 5000 ng/mL in water RTR78464). After vigorous mixing (20 min at 1600 rpm), 200 µL of a water/hexafluoroisopropanol (HFIP)/diisopropylethylamine (DIPEA) solution (100:4:0.2, *v*/*v*/*v*) were added, followed by mixing (15 min at 1500 rpm). Afterward, a clean-up step was performed using solid-phase extraction cartridges (Waters, OASIS HLB, 30 μm) after elution and evaporation to dryness (30–45 min at 40 °C under nitrogen). The samples were reconstituted in 100 µL of water/methanol/HFIP/DIPEA 950/50/5/3.5 (*v*/*v*/*v*/*v*) mobile phase. After vortex mixing (10 min at 1500 rpm), an aliquot (20 μL) was injected into the analytical column (Xbridge Oligonucleotide BRH C18, 2.1 × 50 mm, 2.5 µm (Waters Corporation, Milford, MA, USA)) kept at 60 °C. The analyte and internal standard were separated from matrix interferences using gradient elution from water/methanol/HFIP/DIPEA 950/50/5/3.5 (*v*/*v*/*v*/*v*) to water/methanol/HFIP/DIPEA 100/900/5/3.5 (*v*/*v*/*v*/*v*) within 4 min at a flow rate of 0.4 mL/min. Mass spectrometric detection was carried out on an AB-Sciex 6500+ mass spectrometer using selected reaction monitoring (SRM) in the negative ion mode. The selected ion reactions (*m*/*z*) were 658.8/134.0 for RTR5001 and 670.8/95.0 for RTR78464 internal standard. Detection was accomplished utilizing ion spray MS/MS in negative ion SRM mode. As determined from the analysis of quality control samples, the precision and accuracy of the assay were satisfactory throughout the study. Plasma exposure data were subjected to non-compartmental pharmacokinetic evaluation, and maximum plasma concentration (C_max_) and area under the curve (0–24 h) (AUC_0–24h_) values were determined. One animal from PND 16 was excluded from the exposure assessment analysis due to a sampling error.

#### 2.3.4. Clinical Pathology

Blood samples were used to assess hematology, coagulation, clinical chemistry parameters, and total complement activity (CH50). Additionally, urine was also collected at necropsy and used for urinalysis and urine chemistry assessment. Hematology parameters were determined using an ADVIA 120/2120 system (Siemens, Erlangen, Germany), coagulation parameters were determined with a STA R Max system (Stago, Asnières sur Seine, France), clinical chemistry parameters were determined with an AU680 system (Beckman Coulter, Brea, CA, USA), and CH50 was measured with an in vitro liposome immunoassay CH 50 Autokit (Fujifilm WAKO, Neuss, Germany) on a biochemistry analyzer AU680 system (Beckman Coulter, Brea, CA, USA). For CH50, four treated and four control animals were sampled before RTR5001 administration, 15 min, and 24 h after dosing on each treatment day until PND 28. On PND 36 and 42, four control and only three treated animals were sampled. On PND 50, only two animals per group were sampled. CH50 values were compared with the before-administration values above the lower limit of detection on PND 8, as CH50 levels were not detectable before and after the first dose at PND 1. Urinary chemistry parameters were measured with an AU680 system (Beckman Coulter, Brea, CA, USA). In the results section, only significantly altered parameters when compared with control and/or pre-dosing values are presented.

#### 2.3.5. Necropsy, Anatomic Pathology, Immunohistochemistry, In Situ Hybridization

A complete post-mortem examination was performed, and an extensive list of tissues and organs was fixed and preserved in 10% neutral buffered formalin, embedded in paraffin, sectioned, mounted on glass slides, and stained with hematoxylin and eosin (H&E). The kidney histologic sections were additionally stained with Periodic Acid-Schiff (PAS). Histopathological evaluation was performed for all slides.

Immunohistochemistry. Immunohistochemistry (IHC) to localize ASOs in the kidney (PND 2, 9, 16, 23, 29, 37, 43, and 51), liver (PND 51), and mandibular and retropharyngeal lymph node (PND 2, 43, and 51) samples were performed using a Ventana Discovery Ultra^®^ immunostainer (Ventana Medical Systems, Tucson, AZ, USA). Formalin-fixed and paraffin-embedded tissue sections (3–4 μm thick) of selected animals were deparaffinized, and an anti-ASO pAb2 rabbit polyclonal antibody (synthesized ad hoc by Creative Biolabs) diluted 1:100 was used as a primary antibody (32 min) in a standard protocol using the Ventana Chromo Map DAB^®^ kit (760-159, Ventana). A Discovery OmniMap anti-Rabbit HRP (760-4311, Ventana) was used as a secondary antibody (8 min). No pretreatment was performed. A Discovery Inhibitor (760-4840, Ventana) and S-Block (760-4212, Ventana) (4 min) were selected, and sections were counterstained with hematoxylin.

In situ hybridization. In situ hybridization (ISH) was used to detect and localize RTR5001 in the kidney (PND 2, 9, 16, 23, 29, 37, 43, and 51), liver (PND 51), and lymph node samples (PND 2, 43, and 51). Briefly, tissue sections were deparaffinized and pre-treated with ISH-protease 3 (780-4149, Ventana). Following hybridization with the specific probe, sections were incubated with anti-DIG HRP enzyme conjugate (760-4822, Ventana) in conjunction with a tyramide-based Amplification BF Kit (760-226, Ventana) and anti-BF HRP (760-4828, Ventana). The DISCOVERY Purple kit (760-229, Ventana) was used as chromogen, and specific staining signals were identified as purple punctate dots or diffuse staining present in the cytoplasm. RNA diluent and LNA DIG-labeled U6 probes (provided by Qiagen) were used as negative and positive controls, respectively. Sections were counterstained with hematoxylin II (790-2208, Ventana).

### 2.4. Biobank Study

Snap-frozen liver and kidney samples from different untreated developing and adult female minipigs that were previously collected by Van Peer et al. [49] together with the kidney, liver, and blood samples from four adult males provided by Ellegaard Göttingen Minipig A/S (Dalmose, Denmark), and four adult females provided by Charles River Laboratories France Safety Assessment SAS (Saint-Germain-Nuelles, France) were used in the gene expression and nuclease activity assays for the biobank study. The following age groups were investigated: gestational day (GD) 84–86 and 108; postnatal day (PND) 1, 3, 7, and 28; and adults (aged 14–33 months). GD 84–86 and 108 represent 75 and 95% of gestation length in the minipig, respectively, therefore limiting the fetal age groups to the third trimester of pregnancy. PND 28, which is usually the weaning age in piglets in nonclinical settings, is roughly equivalent to the first year of life in children [42]. Both sexes were equally represented for each tissue and age group except for the kidney samples at PND 3 and 7, and adult kidney and liver samples (Figure 1B).

### 2.5. Gene Expression

The gene expression analysis was first conducted on the biobank samples (*N* = 62), and then, a second analysis was performed on samples from the in vivo study, including biobank adult samples (*N* = 44). The expression profile of seven nuclease genes was assessed and was selected to provide comprehensive coverage of the key endogenous nucleases implicated in ASO metabolism and pharmacologic activity (see Table 1). This included three exonucleases, as ASOs are reported to be degraded by 3′-exonucleases while in circulation and after tissue biodistribution [19,20], and two endonucleases as the digestion of ASOs by these enzymes serve as the initial cleavage event in tissues for modified ASOs [50]. The gene expression profiles of the two isoforms of RNase H in mammalian cells were also evaluated, considering that RNases H hydrolyze RNA in the RNA–DNA hybrids [51]. RNase H1 has been identified as responsible for target RNA degradation in the ASO-driven cleavage mechanism [52]. However, as the definite role of RNase H2 is still unclear, depending on its subcellular localization in specific cell type [53], it was also included in our key nuclease list. Blood samples were evaluated for the relative expression of the exonuclease genes, whereas both exonuclease and endonuclease (both DNase and RNase H isoforms) were evaluated for liver and kidney samples.

Total RNA was isolated using RNeasy^®^ Plus Mini kit (74134, Qiagen, Hilden, Germany) from all liver and kidney samples, and a Nucleospin^®^ RNA Blood Midi kit (740210.20, Macherey-Nagel, Düren, Germany) was used for the blood samples in EDTA-K_2_ anticoagulant following the manufacturers’ instructions. The concentration and purity (OD260/280) of the isolated total RNA were measured directly by UV-Vis Spectrophotometer (NanoDrop Technologies, Wilmington, DE, USA), and the RNA quality was evaluated by running the total RNA in gel electrophoresis, wherein intact rRNA subunits (28S and 18S) were observed, indicating minimal degradation. After extraction, 1 µg of total RNA was reverse transcribed using qScript^®^ cDNA Supermix (95048-500, Quantabio, Beverly, MA, USA) and random hexamers in Q qPCR instrument version 1.0 (Quantabio, Beverly, MA, USA) in a total volume of 20 µL. The first-strand cDNA synthesized was diluted 1/10 with nuclease-free water prior to qPCR.

The primers for the target genes were designed using ApE software v2.0.55 (M Wayne, Madera, CA, USA), ensuring the specificity and inclusion of all transcript variants available on GENBANK and/or ENSEMBL pig sequences. Primers were designed to span different exons to prevent genomic DNA amplification. Primer pair specificities were verified with the Primer-BLAST tool (https://www.ncbi.nlm.nih.gov/tools/primer-blast/, accessed on 1 October 2018), and PCR product sizes were confirmed with gel electrophoresis. Primer sequences and details (i.e., amplicon length, efficiencies) are listed in Table 1. Transcript quantification was performed using a PerfeCTa SYBR Green Fastmix (95072-05K, Quantabio, Beverly, MA, USA) on a Q qPCR instrument version 1.0 (Quantabio, Beverly, MA, USA) in 48-well reaction plates. The qPCR reactions were prepared in a total volume of 20 µL containing 1 µL of cDNA (1/10 dilution), 10 µL SYBR Green Fastmix, and 400 nM for both forward and reverse primers. No-template controls were used for each batch of mixes. The thermocycling program followed a fast 2-step cycling protocol wherein an activation step of 95 °C for 1 min was set, which was followed by 50 cycles of 95 °C for 5 s and 60 °C for 30 s when fluorescence was acquired. A melt curve analysis was generated to check PCR specificity that starts from 72 until 95 °C at a ramp rate of 0.3 °C/s, and wherein single peaks confirmed the specific amplification of the genes. All samples were run in triplicates.

Data were analyzed using the Q-qPCR software v1.0.2 (Quantabio, Beverly, MA, USA). Ct values were used for the analysis of gene expression. Primer amplification efficiencies (E) were determined for each gene in each tissue by calculating the slope of a four-point, five-fold dilution standard curve of a pool of cDNA samples. Gene expression, relative to the most highly expressed sample, was calculated by the ΔΔCt method using reference genes to normalize the expression of target genes. A panel of six commonly used reference genes of which the primer sequences were previously described by Nygard et al. [54] was tested with the geNorm software [55] to evaluate their expression stability among the different age groups, between sexes, and between organs (kidney and liver). Hypoxanthine phosphoribosyltransferase 1 (*HPRT1*) and TATA-box binding protein (*TBP*) were identified as the most stable genes across the different age and sex groups per organ, and they were first used to normalize the data separately for the liver and kidney samples. To allow comparison between kidney and liver data, *HPRT1* and *TBP* were identified to be stable in both the liver and kidney samples for the postnatal age and sex groups, limiting the comparison to postnatal stages. On the other hand, glyceraldehyde 3-phosphate dehydrogenase (*GAPDH*) and *HPRT1* were identified as stable and used to normalize the target gene expression for the blood samples.

### 2.6. Plasma Nuclease Activity

Frozen plasma samples from our biobank and the in vivo study with sodium citrate as anticoagulant were thawed and diluted 1/5 with phosphate-buffered saline (PBS) (pH 7.2). The three isosequential model ASOs were incubated with the plasma in parallel at 37 °C and a final concentration of 20 ng/µL. Five 5 µL-aliquots were taken at time points 0, 15, 30, 60, and 180 min for the unmodified sequence (RTR5001_PO), and 0, 1, 3, 6, and 24 h for the all-PS (RTR5001_PS) and LNA/PS/LNA gapmer (RTR5001). The enzymatic reaction was quenched by adding an equal volume of formamide-containing Gel loading Buffer II (AM8547, Ambion, Oudeschoot, The Netherlands) to the aliquots before storage at −80 °C for further polyacrylamide gel electrophoresis analysis. The samples were thawed and heated at 95 °C for 5 min before the intact and digested oligonucleotides were separated on a 15% denaturing nucleic acid Mini-PROTEAN polyacrylamide gel electrophoresis (4566056, Bio-Rad Laboratories, Hercules, CA, USA) for 50 min at 200 V. Similar to the study of Wahlestedt et al. [56], the gels were subsequently stained with SYBR Gold Nucleic Acid Gel Stain (S-11494, Molecular Probes, Eugene, OR, USA) to directly visualize the oligonucleotides. The gels were visualized and photographed in Gel Doc XR+ System (Bio-Rad Laboratories, Hercules, CA, USA). Densitometric analysis was performed using Image Lab software version 5.1 (Bio-Rad Laboratories, Hercules, CA, USA), wherein the volume density of the major band corresponding to the intact oligonucleotide was calculated in each lane and corrected for background. The volume density of the sample from 0 min/h time point was set as the reference value for each incubation. Plasma nuclease activity was obtained as the percentage of degraded ASO fraction for each incubation time relative to the volume density of the 0 min/h time point. Data from the 15 min (RTR5001_PO) or 1 h (RTR5001_PS, RTR5001) incubation time point was used to evaluate age and sex effects.

### 2.7. Tissue Nuclease Activity

Total protein was extracted from the frozen liver and kidney biobank samples using T-PER Tissue Protein Extraction Reagent (78510, Thermo-Fischer Scientific, Waltham, MA, USA) supplemented with Halt Protease Inhibitor Cocktail (87785, Thermo-Fischer Scientific, Waltham, MA, USA) following the manufacturer’s instructions. Contaminating nucleic acid in the crude tissue extract was precipitated using 2% streptomycin sulfate (S6501-5G, Sigma-Aldrich, Saint Louis, MO, USA), and carry-over streptomycin in the protein extract was removed using Bio-Spin 6 columns (732–6228, Bio-Rad Laboratories, Hercules, CA, USA). The protein content of the tissue extract was determined using a Pierce BCA Protein Assay kit (23225, Thermo-Fischer Scientific, Waltham, MA, USA), and the samples were adjusted to a protein concentration of 1 µg/µl using the T-PER reagent before storage at −80 °C. Incubation of the three isosequential model ASOs with the adjusted tissue protein extract and nuclease activity analysis were as described for the plasma nuclease activity assay. Reaction rates for the liver and kidney homogenates were also computed for the 15 min (RTR5001_PO) or 1 h (RTR5001_PS, RTR5001) incubation time point wherein the relative volume degraded is divided by incubation time.

**Table 1:** Primer design for qPCR, gene details, and PCR efficiencies in the three analyzed tissues: blood (B), kidney (K), and liver (L).

Symbol	Name	Acc. Number	Rationale of Inclusion	Primer (5′–3′)	Amplicon Length	E% (B)	E% (K)	E% (L)
*ENPP1*	Ectonucleotide Pyrophosphatase/ Phosphodiesterase 1	XM_021087933	human orthologue identified as the plasma 3′-exonuclease responsible for PS degradation [20]	(f) CAGATCATGGCATGGAACAAGGCA (r) TGGTTTGGTTCCTGGCAAGAAAG	135	101	103	97
*PDE1B*	Phosphodiesterase 1B	XM_003126207	bovine orthologue was reported to show a similar 3′-exonuclease activity on PS oligonucleotide to that of the human plasma [57]	(f) GACTCGGCACAACCTCATCA (r) CAGTGGACCGTCTGGGTAAC	147	94	100	93
*TREX1*	Three prime repair exonuclease 1	XM_021070628	major and most abundant 3′-exonuclease in mammalian cells [58,59]	(f) CCTGCCTGCTGTTCGGCTC (r) GGCTCTCCAGGGCACATCTAT	175	94	106	98
*DNASE1*	Deoxyribonuclease 1	NM_213991	well-characterized role in DNA degradation [60,61]	(f) GGGATCTGGAGGACATCATGCT (r) CGACCACGATCCTGTCATAGGC	177	-	97	88
*DNASE2*	Deoxyribonuclease 2	NM_214196	well-characterized role in DNA degradation [60,61]	(f) GGAGGAGGTAGTCAAGGGCCA (r) GCCAGAGTACAGGTCGTCTCC	133	-	90	97
*RNASEH1*	Ribonuclease H1	NM_001243681	demonstrated its role for ASO pharmacologic activity [52]	(f) GCCAGGCCATCCTTTAAATGTAGG (r) CCCAGCTAGTGATGCCATTGATGG	170	-	98	96
*RNASEH2A*	Ribonuclease H2 subunit A	NM_001244444	isoform of ribonuclease H in mammalian cells, capable of degrading target RNA in cell lysates [8,53]	(f) TTTGTGGGCTGGGCATTGGA (r) ACAAACACCTGGGCCACTTTC	158	-	101	89

### 2.8. Statistical Analysis

To evaluate the effect of age and sex on nuclease gene expression and activity, data on the biobank liver and kidney samples were fitted first to a linear mixed model. The fixed factors of the model for this analysis consisted of age and sex, together with their interaction. Then, a second analysis for the in vivo study samples was performed for genes that did not have sex or age–sex interaction on the initial analysis. Age was the only fixed factor included in the model for the second analysis. Treatment as an effect was not included, as it is not expected to affect nuclease gene expression. To account for the dependence between observation among littermates, sow was set as a random effect on the model. Run-by-plates (gene expression) or run-by-gel (activity assay) were added as a random factor to the model to correct for inter-run variability. The starting model was gradually simplified using stepwise backward modeling, wherein all non-significant effects were removed step by step. Post hoc analysis with Tukey’s honest significance test was used when comparing the different age groups. When an age–sex interaction was detected, the effect of age was evaluated separately for both sexes. To evaluate the difference in gene expression and nuclease activity reaction rates between the kidney and liver samples per age group, organ, sex, and their interaction were used as fixed factors in the model. A non-parametric Spearman rank correlation test was performed to identify the correlation between nuclease gene expressions and activity toward the isosequential ASOs and between the exposure parameters and mean plasma albumin concentration for the investigational toxicity study. For the other in vivo parameters, no statistical analyses were performed due to limited sample size, except for the complement activity. To evaluate the effect of age, treatment, and their interaction on complement activity, data were fitted to a linear mixed model with Dunnett’s multiple comparison test using PND 8 control (predose value) as reference. The model included age, treatment, and their interactions as fixed factors. Animals nested into the treatment group were set as a random effect to account for repeated measures for each subject. A p-value smaller than 0.05 was considered statistically significant. Variables were log- or square-root transformed when needed to meet normality and/or homoscedasticity assumptions. Statistical analysis and graphs were done using JMP^®^ Pro 15 (SAS Institute, Cary, NC, USA) and GraphPad Prism 8 (La Jolla, CA, USA).

## 3. Results

### 3.1. Exposure Assessment and Tissue Biodistribution

**Figure 2:**
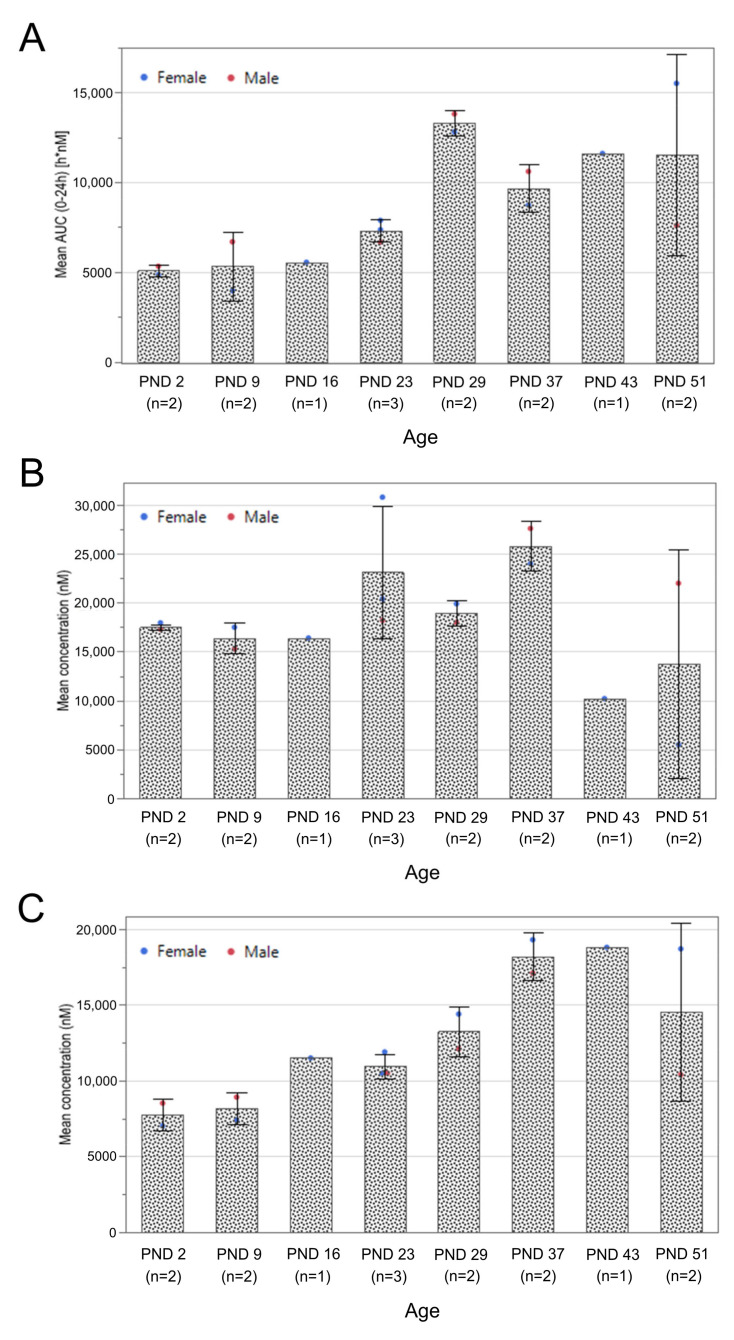
Mean ± SD exposure levels in the (**A**) plasma, (**B**) kidney cortex, and (**C**) liver after repeated subcutaneous administration of RTR5001 to different developing minipigs at 20 mg/kg dose level. The exposure in plasma (bound and unbound proportion) was measured as AUC of plasma concentration over a time interval of 0 to 24 h.

Based on the limited number of treated animals per group, only descriptive results for the temporal trends of RTR5001 concentrations in the plasma, kidney, and liver are presented. For the plasma AUC_0–24h_ and C_max_, comparable values after the first four subcutaneous administrations (PND 2, 9, 16, and 23) were observed (Figure 2A and Figure S1). A relatively higher AUC_0–24h_ value at PND 29 was seen after the fifth dosing. This was followed by a slight decrease that remained relatively unchanged from the sixth until the eighth dose (PND 37, 43, and 51). In contrast, C_max_ increased gradually after the fifth dose. The highest C_max_ value was reached after the last dose, and it was 2.3-fold higher than the C_max_ after the initial dose at PND 1. After 24 h post-administration, RTR5001 was rapidly cleared from the blood circulation, resulting in concentrations two to three orders of magnitude lower than the C_max_. Plasma trough levels measured directly before RTR5001 administration were relatively low at PND 1, 8, 22, 28, and 36, whereas slightly higher concentrations were seen at PND 15, 42, and 50 (Figure S1).

The RTR5001 concentration in the kidney (Figure 2B) was steady following the first three dosing days (PND 2, 9, and 16). Afterwards, a higher concentration was observed through the fourth to sixth doses (PND 23, 29, 37), which was followed by a drop in concentration by about half for the seventh (PND 43) and eighth (PND 51) doses. In contrast, a gradual increase in concentration of RTR5001 was observed in the liver, reaching a plateau between the sixth and seventh doses (PND 37 and 43) (Figure 2C), which was followed by a lower concentration after the final dose (PND 51). Generally, the compound distributed more in the kidney than in the liver except at PND 43. Spearman’s rank correlation analysis on the three exposure panels showed a high correlation between plasma AUC_0–24h_ and the liver exposure levels (*p* < 0.0001, *r* = 0.8436, *n* = 15) but failed to detect a significant correlation between plasma AUC_0–24h_ and the kidney exposure levels (*p* = 0.8101, *r* = 0.0679).

### 3.2. In-Life Observation and Clinical Pathology

All animals survived up to the scheduled humane killing, and RTR5001 was clinically well-tolerated at 20 mg/kg/dose. There were no treatment-related effects on the physical development of piglets (i.e., pinna unfolding, eye opening, and incisor eruption), body weight, and no injection site reactions were observed for any RTR5001-treated animals.

**Figure 3:**
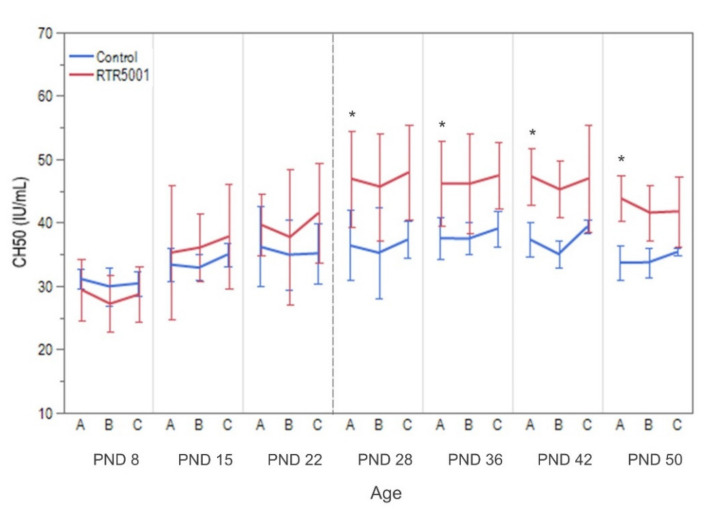
Mean ± SD total complement (CH50) activity measured in the serum of control and RTR5001-treated minipigs in different juvenile age groups: pre-dose (A), 15 min post-dose (B), and 24 h post-dose (C) of RTR5001. The same animals were tested (control: *n* = 4; RTR5001-treated: *n* = 4) from PND 1 until PND 28, and reduced at PND 36 and 42 (control: *n* = 4; RTR5001-treated: *n* = 3), and PND 50 (control: *n* = 2; RTR5001-treated: *n* = 2) accordingly. Broken line indicates weaning time (at PND 28) for the piglets. Differences between age, experimental groups, and their interactions before RTR5001 administration were determined using a mixed model with Dunnett’s multiple comparison tests. *p* value < 0.05 when compared to PND 8 control (pre-dose value) was considered significant (*). CH50 was not significantly reduced 15 min post-RTR5001 administration within each age group, but it was significantly higher for the pre-dose values of PND 28 until 50 compared to the PND 8 control group after repeated administration of RTR5001.

The administration of RTR5001 led to a minimal to mild increase in white blood cells (neutrophils and lymphocytes) and a minimal to mild increase in fibrinogen. No changes for coagulation parameters were noted (see Table 2). Moreover, a mild increase in aspartate aminotransferase (AST) was observed, but no effect was seen on urinalysis or urine chemistry parameters (see Table 2). The total complement activity measured by CH50 was not detectable before and after the initial dosing at PND 1. Activity levels on PND 8 until PND 22 between control and treated animals were comparable. However, RTR5001 caused a significantly increased CH50 on the pre-dose values from weaning onwards compared to PND 8 control group (*p* = 0.0364), and it did not differ statistically over time (*p* = 0.9397). On the other hand, there was no significant change in total complement activity 15 min post-RTR5001 administration within each age group (Figure 3).

**Figure 4:**
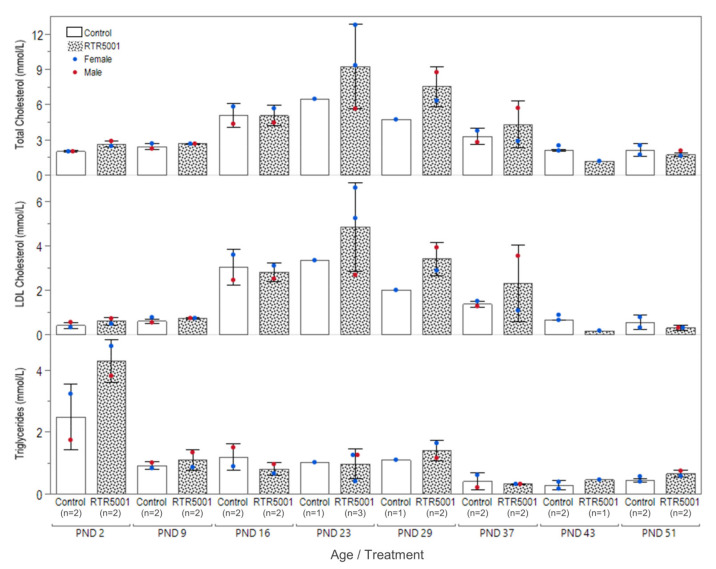
Mean ± SD level of serum total cholesterol, LDL cholesterol, and triglycerides in control and RTR5001-treated minipigs in the different juvenile age groups.

The serum total cholesterol and low-density lipoprotein (LDL) cholesterol levels of both control and treated groups remained stable from PND 2 to PND 9 (Figure 4). Both parameters gradually increased until PND 23 before gradually decreasing back to the basal level again on PND 43 and 51. In contrast, a peak triglyceride level was observed at PND 2 before an initial drop on PND 9 that stayed stable until PND 29 (Figure 4). Another drop could be observed at PND 37 and remained stable until PND 51. No overt decrease in the three parameters (total cholesterol, LDL cholesterol, and triglycerides) was observed in treated groups after the first six weekly doses. In contrast, values for both LDL- and total cholesterol panels in the treated animals were higher at PND 23, 29, and 37 compared with the controls. However, when the total cholesterol values were compared with published control data in Göttingen Minipigs [62], only values at PND 23 were above the normal range. Afterwards, decreases on PND 43 and PND 51 for total cholesterol (44% and 19% lower, respectively) and LDL cholesterol (74% and 44% lower, respectively) were seen for the treated animals when compared with control animals in the study and with previous data [62].

**Figure 5:**
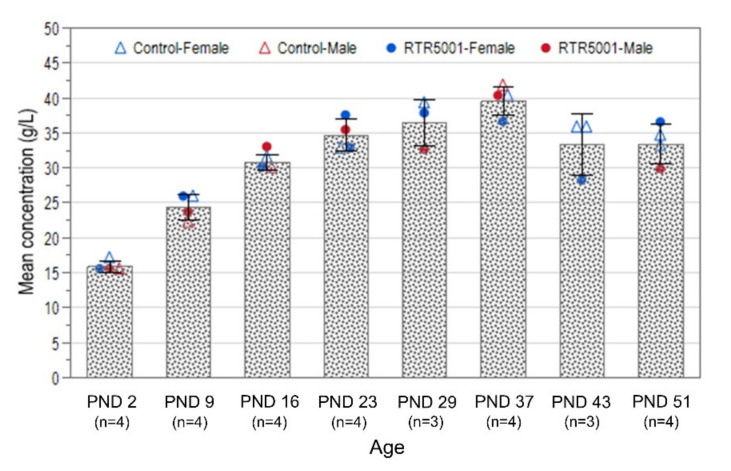
Mean ± SD plasma albumin concentration in control and RTR5001-treated juvenile minipigs in the different juvenile age groups.

No treatment-related effects were observed in the mean plasma albumin concentration, but an age-related effect was observed (Figure 5). A gradual increase in both control and treated groups was observed, reaching the highest concentration at PND 37. A slight decrease was observed at PND 43, which remained unchanged at PND 51. Spearman’s rank correlation analysis showed high correlations between the mean plasma albumin concentration and AUC_0–24h_ (*p* < 0.0121, *r* = 0.6286, *n* = 15), kidney exposure levels (*p* < 0.0218, *r* = 0.5857, *n* = 15), and liver exposure levels (*p* < 0.0055, *r* = 0.6774, *n* = 15).

### 3.3. Anatomic Pathology, Immunohistochemistry, and In Situ Hybridization

The repeated subcutaneous administration of RTR5001 did not cause any relevant gross lesions except at the injection sites. After microscopic evaluation, RTR5001-related histopathological findings were observed in the kidney, lymph nodes, and injection sites.

**Figure 6:**
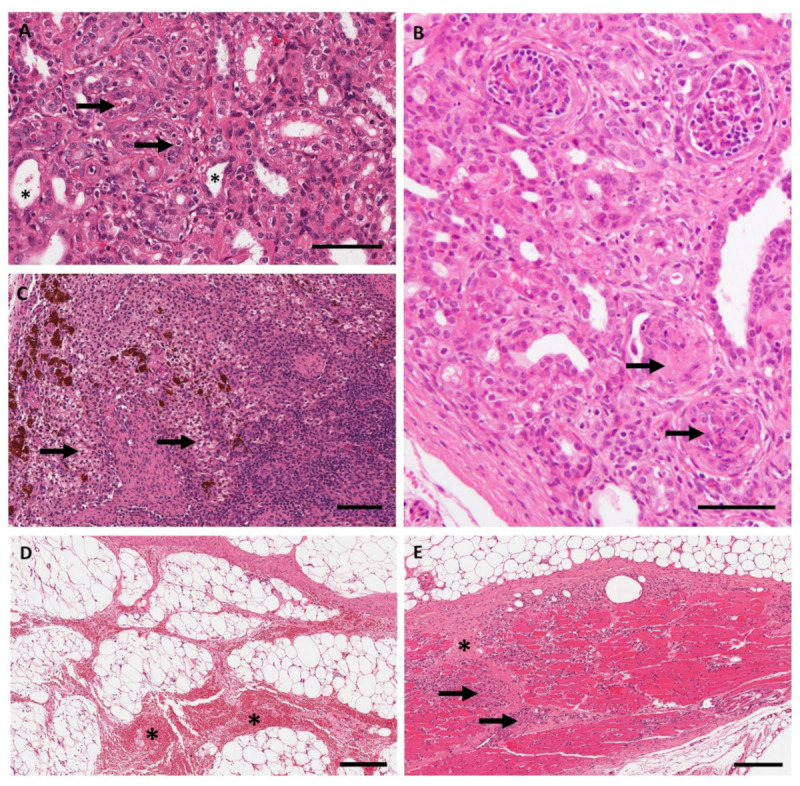
Histopathology findings. (**A**) Kidney, PND 43, H&E; Necrosis of tubular cells (arrow), tubular degeneration (asterisk). (**B**) Kidney, PND 51, H&E; Interstitial fibrosis and inflammatory cell infiltration, and glomerulosclerosis (arrow), (**C**) Lymph node, PND 51, H&E; Foamy macrophages (arrow) and brown pigment in the sinus macrophages due to intramuscular iron injection. (**D**) Injection site, PND 37 (control), H&E; Hemorrhage (asterisk) in the subcutaneous adipose tissue. (**E**) Injection site, PND 43 (treated), H&E; Inflammation (arrow) and fibrosis (asterisk) of the panniculus muscle. (Scale bar: (**A**–**C**) = 100 µM; (**D**,**E**) = 200 µM).

In the kidneys (Figure 6A,B), RTR5001-related minimal tubular degeneration/regeneration was observed at PND 23 onwards. At PND 43 and 51, the tubular changes were minimal to mild in severity and were accompanied by mononuclear cell infiltration, glomerulosclerosis, fibrosis, basophilic granules, and hyaline casts in the renal cortical region. Only scattered and barely visible basophilic granules considered to reflect the oligonucleotide uptake were observed in the cytoplasm of the epithelial cells lining the renal tubules at PND 51. 

In the lymph nodes (Figure 6C), foamy/granular macrophages were observed in the retropharyngeal lymph node at PND 37, and in the mandibular, mesenteric, and superficial cervical lymph nodes at PND 43 and 51. There were no changes in the lymphoid tissue of these lymph nodes. Brown pigment consistent with iron deposits was observed in the examined lymph nodes.

At the injection sites, gross and microscopic findings were at a higher incidence in the treated groups (69%) than in the control (21%) animals. Dark foci were observed in control and treated animals, and they were correlated microscopically with dermal and/or subcutaneous inflammation and hemorrhages, which were accompanied by inflammation and/or degeneration of the panniculus muscle (Figure 6D,E). The severity of the inflammatory changes was slightly higher (minimal to mild) in treated piglets at PND 43 and 51 compared to the other sacrifice time points where the severity was generally minimal. In control animals, the inflammation was acute, and no fibrosis was observed. No basophilic granules or foamy/granular macrophages were observed at injection sites.

**Figure 7:**
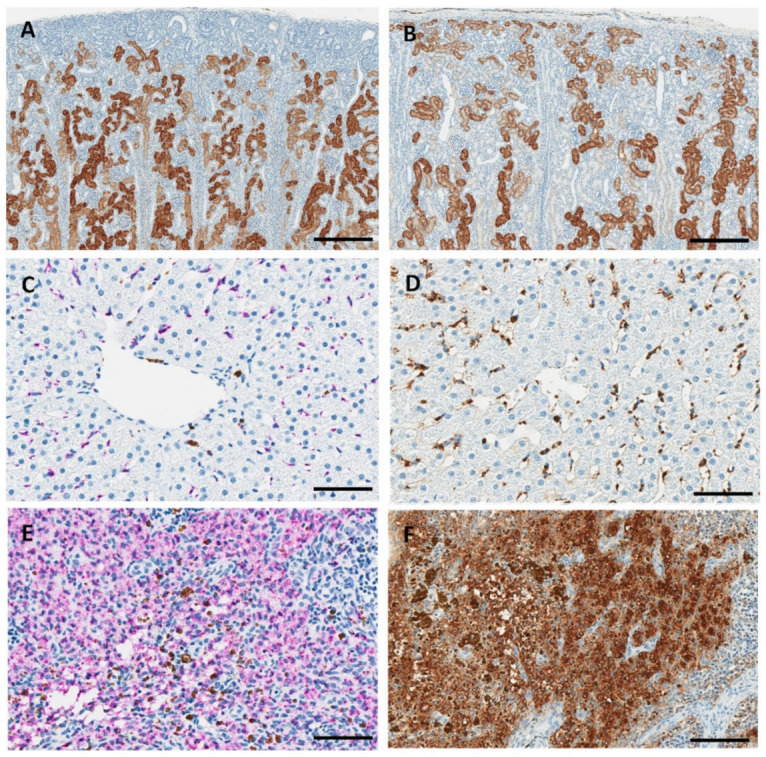
Immunohistochemistry and in situ hybridization. (**A**) Kidney, PND 2; immunohistochemistry for RTR5001. Accumulation of RTR5001 into the proximal tubular cells (brown staining). Renal tubular cells of the outer cortex below the capsule are not stained. (**B**) Kidney, PND 37; immunohistochemistry for RTR5001. Accumulation of RTR5001 in the tubular cells including the outer cortex. (**C**) Liver, PND 51; in situ hybridization for RTR5001. Accumulation of LNA into the Kupffer cells (purple staining) and brown pigment (iron deposits). (**D**) Liver, PND 51; Immunohistochemistry for RTR5001. Presence of brown staining due to accumulation of LNA in Kupffer cells and presence of iron pigment deposits cannot be differentiated, unlike with ISH-stained sections (image C). (**E**) Lymph node, PND 43; in situ hybridization for RTR5001. Accumulation of RTR5001 into the vacuolated macrophages (purple staining) and brown pigment (iron deposits) in sinus macrophages. (**F**) Lymph node, PND 43; immunohistochemistry for RTR5001 Accumulation of RTR5001 and presence of iron pigment deposits. (Scale bar: (**A**,**B**) = 100 µM; (**C**–**F**) = 50 µM).

Immunohistochemistry and in situ hybridization revealed the presence of RTR5001 in the renal tubular cells, Kupffer cells, and lymph node macrophages of juvenile minipigs treated with RTR5001 (Figure 7).

Immunohistochemistry for RTR5001 demonstrated pronounced positive cytoplasmic staining in the renal cortical tubular cells already after the first administration of RTR5001, and there was no apparent increase over time. In the youngest minipigs (PND 2, 9, and 16), the renal tubules in the outer cortex below the capsule were not stained (Figure 7A,B). This area contains smaller and immature nephrons that lack differentiation, and therefore may not yet be functional at that age [63]. In the animals with the highest degree of tubular degeneration/regeneration at histopathological evaluation of the H&E sections (PND 23, 43, and 51), fewer tubules were stained, and the staining was irregularly distributed compared to the non-affected kidneys (Figure S2). Positive staining was characterized by brown granules in the cytoplasm of tubular cells. In situ hybridization for RTR5001 demonstrated positive cytoplasmic reaction in the renal tubular cells. Positive staining was characterized by purple, punctate dots and diffuse staining in the cytoplasm of tubular cells.

In the liver, positive staining in Kupffer cells was observed by ISH (Figure 7C) and IHC (Figure 7D), as evident by purple and brown pigments, respectively. However, the brown positive stain was partially due to intramuscular administration of iron, which is routinely done in newborn minipigs to prevent iron-deficiency anemia [64].

In the lymph nodes (mandibular and retropharyngeal), positively stained macrophages in the sinus were observed in both IHC- and ISH-stained slides (Figure 7E,F). This corresponded to the presence of foamy/granular macrophages observed in the lymph node sinus at histopathological evaluation of the H&E sections. Similar to the liver, brown pigments consistent with iron-containing hemosiderin deposits were observed in the sinus macrophages of lymph nodes stained by ISH. In IHC, this pigment could not be distinguished from the brown DAB staining.

### 3.4. Ontogeny of Nuclease Gene Expression and Activity in the Blood

**Figure 8:**
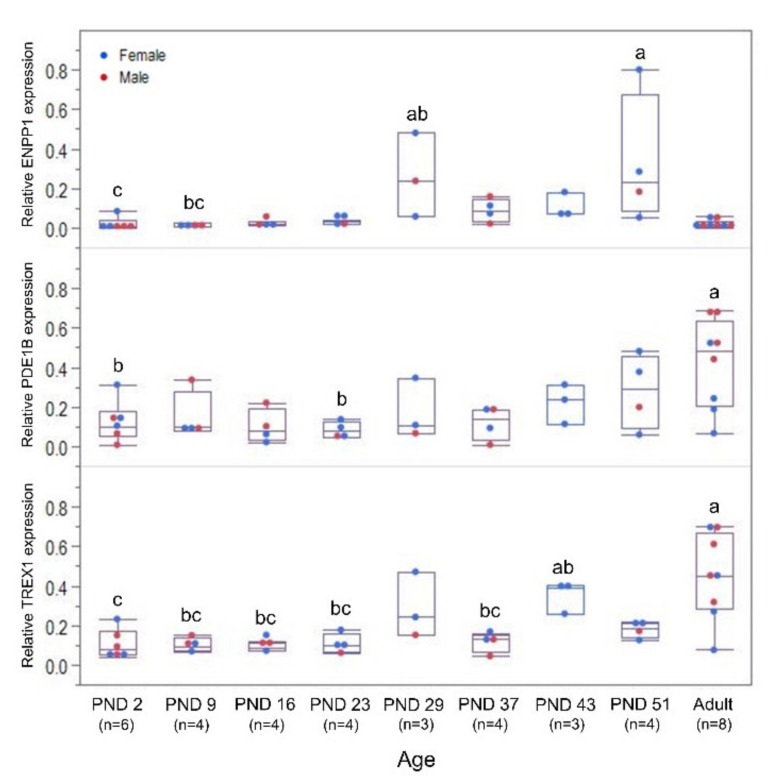
Relative gene expression of 3′-exonucleases: *ENPP1*, *PDE1B*, and *TREX1* in the blood over time in the developing and adult Göttingen Minipig. Age groups not sharing the same letter within each gene panel show significantly different gene expression (*p* < 0.05).

Low expression of the three 3′-exonuclease genes was observed in the blood derived from the youngest age groups from the in vivo study (Figure 8). *ENNP1* expression level showed a transient mild increase at PND 29, and the highest level was reached at PND 51 (*p* = 0.0002). Afterwards, *ENNP1* expression dropped to the same level as the youngest groups. This is in contrast to the *PDE1B* expression, which continued to increase and reached the highest level in adulthood (*p* = 0.0176). The highest *TREX1* expression level was also observed at the adult group (*p* = 0.0001), but no clear maturation profile was observed for the older juvenile stages.

**Figure 9:**
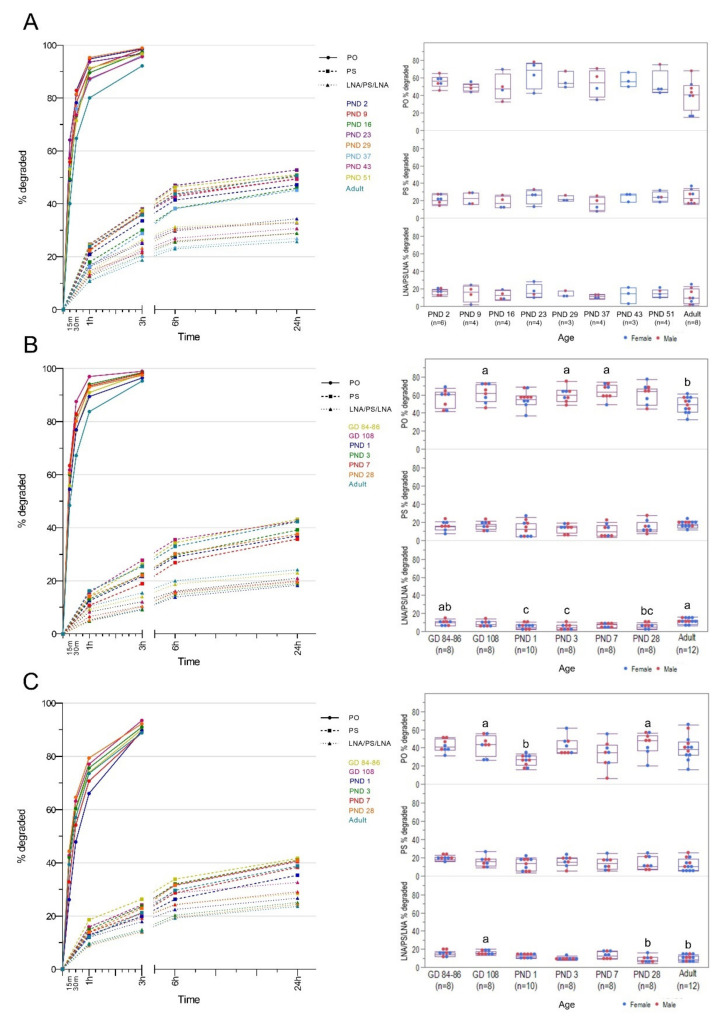
Degradation of isosequential ASOs: unmodified (PO), all-PS (PS), and LNA/PS/LNA gapmer (RTR5001) in (**A**) plasma, (**B**) kidney, and (**C**) liver over time in the developing and adult Göttingen Minipig. Nuclease activity is represented by the percentage of degraded ASO fraction for each incubation time on the left panel: PO (0, 115, 30, 60, 180 min), PS and LNA/PS/LNA (0, 1, 3, 6, 24 h). The rate of ASO degradation for the two modified sequences slowed down between 6 and 24h of incubation. The degraded ASO fraction percentages at the 15 min (PO) and 1 h (PS, LNA/PS/LNA) time points between different age groups are presented in the right panels. Age groups not sharing the same letter are significantly different (*p* < 0.05).

Regarding nuclease activity, the unmodified (PO) ASO was almost completely metabolized in the assay within three hours (Figure 9A, left panel), whereas the two modified ASOs exhibited stability against the endogenous nucleases in the plasma. This was markedly observed with the LNA/PS/LNA gapmer and, to a lesser degree, with the all-PS after 24 h of incubation. When looking at the degradation of the different ASOs after 15 min (PO) or 1 h (PS, LNA/PS/LNA) incubation with plasma, no statistically significant difference between the age groups was detected (PO: *p* = 0.6323; PS: *p* = 0.0507; LNA/PS/LNA: *p* = 0.8182) (Figure 9A, right panel).

### 3.5. Ontogeny of Nuclease Gene Expression and Activity in the Kidney and Liver

**Figure 10:**
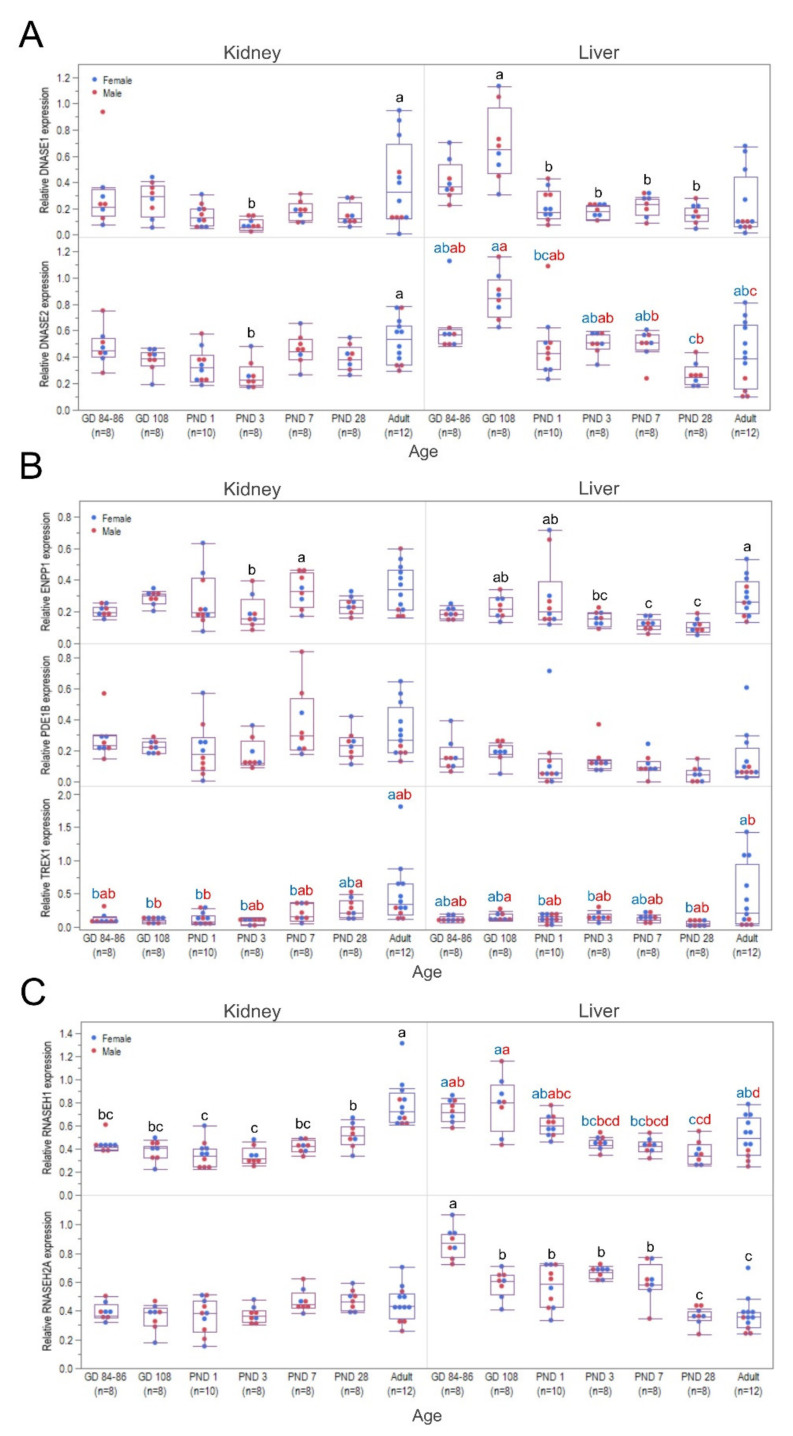
Relative gene expression of nucleases for ASO metabolism and pharmacologic activity over time in the developing and adult Göttingen Minipig. Endonucleases: *DNASE1* and *DNASE2* (**A**); 3′-exonucleases: *ENPP1*, *PDE1B*, and *TREX1* (**B**); and *RNASEH1* and *RNASEH2A* (**C**) were evaluated in kidney and liver. Age groups not connected by same letter are significantly different (*p* < 0.05). When sex–age interaction was detected, the gene expression differences between age groups were evaluated separately for females and males. Same colored letters belong to the same sex, and age groups not sharing the same letters are significantly different (*p* < 0.05).

In general, higher expression of the endonuclease and exonuclease candidate genes were observed in kidney than in liver samples from our biobank (Figure S3). A similar ontogeny profile was observed for both members of the DNase endonuclease family in the kidney, in which they exhibited a significantly lower expression at PND 3 than in the adult group (*DNASE1*: *p* = 0.0046; *DNASE2*: *p* = 0.0387; Figure 10A, left panel). A gradual increase was observed after PND 3 and eventually reached the highest levels at the adult stage. In contrast, liver *DNASE1* exhibited a significantly higher expression at GD 108 than the postnatal age groups, with GD 84–86 and adults showing intermediate values (*p* = 0.0112). A downregulation after birth was observed for liver *DNASE1* expression, and it remained the same until PND 28. Moreover, a sex–age interaction was detected in liver *DNASE2* expression (*p* = 0.0001); i.e., males exhibited a gradual decrease from PND 1, reaching the lowest level at the adult stage (*p* = 0.0001). In contrast, expression in females reached the lowest level at PND 28, before increasing again in adulthood (*p* = 0.0001).

The 3′-exonucleases in the kidney and liver presented ontogeny profiles that were different from the ones of the endonucleases (Figure 10B). Kidney *ENPP1* showed its lowest expression at PND 3, which was followed by an increase to its highest level at PND 7, while the other age groups showed intermediate values (*p* = 0.0142). Liver *ENPP1* remained lowly expressed from PND 3 until PND 28 before increasing to a higher level in adulthood that was comparable to the expression at the fetal stages until PND 1 (*p* = 0.0001). On the other hand, *PDE1B* expression remained unchanged throughout development in both kidney and liver (*p* = 0.0581; *p* = 0.0720, respectively). A sex–age interaction in *TREX1* expression was detected in both organs (kidney: *p* = 0.0299; liver: *p* = 0.0001, respectively). Kidney *TREX1* expression in males exhibited a higher level at PND 28 than in the perinatal stages, with other age groups showing intermediate values (*p* = 0.0117). Kidney *TREX1* expression remained low for the first week of life in females, before gradually increasing until adulthood (*p* = 0.0001). Similarly, a higher expression of *TREX1* was seen in the adult female liver samples (*p* = 0.0001). Meanwhile, a lower liver *TREX1* expression in adult males was observed compared to GD 108, with the other age groups showing values in between (*p* = 0.0295). These observed ontogeny profiles in the biobank samples are mostly in congruence with the expression patterns seen in the samples from the in vivo study (Figure S4A,B).

The stability of the two modified isosequential ASOs and marked susceptibility of PO ASO to nuclease activity in the kidney and liver homogenates were similar to what was observed in the plasma (Figure 9B,C, left panels). However, reaction rates toward the PO ASO in the kidney homogenates were significantly higher than in the liver except in the adult group (Figure S5). The rate of PS ASO degradation was observed to be higher in the liver at GD 84–86, whereas it was seen higher in the kidney for the adult group. The opposite observations for PO ASO were seen in the LNA/PS/LNA gapmer, wherein degradation generally proceeded faster in the liver of the fetal and juvenile age groups. A relatively higher activity toward PO ASO was observed at GD 108 and PND 3–7 in the kidney (*p* = 0.0001), while higher activity was seen at GD 108 and PND 28 in the liver (*p* = 0.0156) (Figure 9B,C, right panels). On the other hand, lower activity in the kidney was observed in the adult group, whereas it was detected immediately after birth in the liver homogenates. No statistically significant difference was observed among the different age groups in terms of activity toward the isosequential all-PS ASO (kidney: *p* = 0.4758; liver: *p* = 0.3568). Degradation of the LNA/PS/LNA gapmer in the kidney was slower at PND 1–3, but it gradually accelerated until adulthood (*p* = 0.0001). In contrast, higher activity in the liver was seen at GD 108, and a lower activity was seen at PND 28 and in the adult group (*p* = 0.0033). Spearman’s rank correlation analysis on all age groups together showed a moderate correlation between liver DNASE1 expression and the activity toward the LNA/PS/LNA gapmer in the liver (*p* < 0.00026, *r* = 0.4175).

### 3.6. Ontogeny of Ribonuclease H expression in the Kidney and Liver

Kidney *RNASEH1* expression showed a postnatal maturation profile reaching the highest level in adulthood (*p* = 0.0001). No maturation profile was noted for kidney *RNASEH2A* (*p* = 0.1458). In contrast, the liver expression of the two ribonuclease Hs displayed a gradual decrease postnatally (Figure 10C). A sex–age interaction was detected in the expression of *RNASEH1* in the liver (*p* = 0.0011). Although both males and females showed lower expression at PND 3 than in the gestational period, this lower expression was detected until PND 28 (*p* = 0.0001) and then increased in adulthood in females, reaching similar values to those in the youngest age groups. In contrast, the lower transcription at PND 3 than during gestation in males remained unchanged until adulthood (*p* = 0.0001). For both male and females, liver *RNASEH2A* expression showed the highest expression values on GD 84–86, an initial drop in expression on GD 108, and a second one on PND 28 (*p* = 0.0001). In general, these ontogeny profiles in the biobank samples are in accordance with the expression patterns seen in the in vivo study samples (Figure S4C).

## 4. Discussion

In the present study, the safety, exposure, and pharmacological effect of a model LNA gapmer, RTR5001, were assessed in a limited number of juvenile Göttingen Minipigs after repeated subcutaneous dosing. The rapid growth and development of the pediatric population can influence the pharmacokinetics and pharmacodynamics of therapeutic agents, leading to potential adverse effects [65,66,67]. RTR5001 was previously tested in adult minipigs, in which the liver and kidney were the main target organs of distribution/toxicity, and its pharmacologic effect (i.e., reduced total and LDL cholesterol) was demonstrated [31]. Similar clinical chemistry and toxicity findings were observed in the juvenile minipigs in our study. However, differences in plasma and tissue exposure, as well as pharmacologic activity, were seen compared with the adult data. To elucidate these findings, the expression and activity of nucleases involved in ASO metabolism and pharmacologic activity were assessed in the blood, kidney, and liver of juvenile Göttingen Minipigs. For clarity, we will discuss our results following the order in which ASOs interact in the animal. First, we will discuss the metabolism and exposure in the blood compartment, followed by distribution, metabolism, and finally toxicities in the tissue compartments. The remainder of the discussion will be devoted to other relevant toxicities and the pharmacologic effect of the model LNA gapmer.

The nuclease activity profiles in the blood and tissue compartments were assessed using isosequential PO, all-PS, and RTR5001 (LNA/PS/LNA gapmer) ASOs. Our findings for the three isosequential ASOs were similar in the blood and tissue compartments, with RTR5001 showing excellent stability for nucleases, followed by all-PS, and with the PO sequence being very susceptible to degradation. This is in accordance with previous results in adult rodents, NHPs, and humans for the blood compartment [56,68], liver [69,70,71], and kidney [72]. PO ASOs are degraded within minutes in the blood and tissue compartments by endogenous nucleases, and thus, chemistry modifications of the phosphate bonds and sugar moiety of the oligonucleotide sequence are warranted to improve nuclease stability [6,22,73,74,75]. In addition to ASO chemistry modifications, sequence length also influences the rate of metabolism, as shorter sequences are digested more slowly [71]. Therefore, initial degradation products from nuclease digestion compete with full-length sequences and contribute to their stability [76]. This can influence the rate of nucleolytic metabolism in vitro, as shown by the plateau in our activity assay between 6 and 24 h incubation.

For the blood compartment, no age-related differences in nuclease activity were observed for any of the investigated ASOs. 3′-Exonuclease is the main nuclease that acts on PS-modified ASOs in the blood compartment similar to PO [77], with the former having more significant enzymatic stability due to its backbone modification [77,78,79,80]. Differences in the ontogeny profile of the three 3′-exonucleases regarding their gene expression were observed. Among those three nucleases, ENPP1 is secreted in the plasma [81] and has been implicated in the degradation of ASOs in adult human plasma [20]. In contrast, PDE1B and TREX1 are localized mainly intracellularly [58,82], which suggests that they generally play a role in ASO metabolism in white blood cells and tissues. Although we have seen differences in *ENPP1* expression among different age groups, this did not translate to differences in activity in plasma. As discrepancies between RNA and protein abundance have been described before [83,84], mRNA is not always a good indicator of activity [85,86]. On the other hand, the additional flank modifications using other generations of chemistry modification, such as LNA leading to a gapmer design, provide additional/sufficient protection against exonucleases. Endonucleases can degrade LNA gapmers but do this over a more extended incubation period [56,68] in the plasma. However, due to increased plasma protein binding and excellent biodistribution properties of gapmer ASOs [87,88,89], they are quickly distributed to target organs. In the case of RTR5001, these are the kidneys and the liver. Thus, the main factor for plasma clearance is the initial rapid tissue distribution [87,90] instead of ASO metabolism.

The rapid elimination of RTR5001 from the circulation, i.e., within 24 h post-administration, was similar to the data in adult minipigs [31]. The short distribution half-life in the plasma of around 2–5 h for the various juvenile age groups complied with data on second-generation ASOs [91,92]. The biodistribution of ASOs from the plasma to other tissue compartments is related to plasma protein binding as well as to cell-surface proteins in the target organs [87,93,94,95,96], with plasma albumin being the main plasma protein responsible for protein binding in the blood compartment [87,97,98,99,100], preventing renal clearance [76,101]. In our study, plasma albumin concentrations in the juvenile age groups were in accordance with already published reference values, and they were 10 to 60% lower than in 6-month-old minipigs [62]. Therefore, the lower plasma albumin concentration observed in the younger age groups could partly explain the lower C_max_ and AUC_0–24h_ values seen at PND 2 to 23 compared to the adult minipig (Figure S6A,B) [31]. Of note, the juvenile minipigs were dosed up to eight times at a weekly interval, whereas all the adult minipigs were dosed only four times with a 5-day interval. After the first administration, the C_max_ of RTR5001 was 43% lower in juvenile (at PND 2) minipigs than in adult minipigs, whereas the AUC_0–24h_ was 78% lower. After four doses, these values were still lower in juvenile compared to the adult minipigs. As the peak levels of ASOs do not tend to increase with repeated administration due to their wide tissue distribution [88,102,103], no differences were observed between the first and fourth dose. However, after the eighth and final dose in the juvenile minipigs, both parameters gradually increased, with C_max_ being 29% higher than the adult values (after four doses), whereas the AUC_0–24h_ was still 50% lower than the adult values. This lower AUC_0–24h_ could be due to a faster renal clearance of RTR5001 in the juvenile age groups than in the adult. On the other hand, the plasma trough levels after each weekly dose remained relatively very low, which is in contrast to the behavior in older juvenile age groups where at the end of the experimental period, trough levels were slightly higher and indicative of tissue saturation, as has been described before for ASOs after repeated administration [16,21,50,101,104,105].

The highest concentrations of RTR5001 were seen in the kidney and liver and were similar in the various juvenile age groups, with the kidney showing higher exposure than the liver. This has been demonstrated for the biodistribution of LNA gapmers [12,106,107]. However, the relative kidney exposure in the juvenile minipigs was less than the 5-fold difference observed in the adult minipig after the fourth dose (Figure S6C) [31]. This could be explained by the use of different bioanalytical methods. While in our study, LC-MS/MS was used, for the published adult minipig exposure data, a hybridization enzyme-linked immunosorbent assay (hELISA) was used to measure the tissue concentrations of RTR5001. Although this method has good sensitivity to the parent compound, it can cross-react with 3′- and 5′-end putative metabolites [108,109,110]. As we noted that RTR5001 was metabolized at a higher rate in the adult kidney homogenate than in the younger age groups, the higher concentration in the adult kidney could be a cumulative concentration of the parent RTR5001 and its metabolites resulting from the relatively unspecific hELISA.

After repeated dosing, there was no apparent accumulation of the ASO [16,104] in the kidney. The presence of RTR5001 was confirmed in the cytoplasm of renal tubular cells by ISH and IHC immediately after the first dose, and it was similar with successive dosing. This stable exposure observed in the youngest age groups (PND 2, 9, and 16) might be due to the immaturity of the renal cortex [63] resulting in reduced uptake of ASOs. This finding is in line with current knowledge that completion of nephrogenesis occurs at PND 21 in pigs [111]. In contrast, the lower exposure values observed at PND 43 and PND 51 could be due to the high degree of tubular degeneration/regeneration and minimal to moderate renal fibrosis and glomerulosclerosis observed in these age groups. Hence, these injured tubules might have limited capacity to take up LNA ASOs further. Moreover, the distinct basophilic cytoplasmic granules in renal tubular cells previously seen in the adult minipig [31] and other species [13,112,113] were observed only at PND 51. The administration of RTR5001 resulted in degenerative/regenerative changes in the kidneys, similar to what was observed in adult minipigs [31]. However, the glomerulosclerosis seen in minipigs at PND 43 and 51, which received seven and eight doses respectively, was not previously observed in the adult minipigs that were dosed only four times. Despite these histological findings, clinical chemistry did not suggest a decrease in kidney function in the juvenile minipigs as opposed to what was observed in the adult minipig.

For the liver, an increasing exposure was observed after repeated dosing, suggesting accumulation. This coincided with the highest plasma trough level in the two oldest age groups, suggesting higher tissue accumulation than in the younger age groups. ISH and IHC confirmed the accumulation of RTR5001 in the liver, i.e., in the Kupffer cells. However, no distinct basophilic cytoplasmic granules were seen, unlike in the adult minipig and in other species [13,31,112,113]. Furthermore, in contrast to the adult minipig, no hepatocellular single-cell necrosis/apoptosis was seen, although a minimal to mild increase in serum transaminase was observed in some age groups, indicating liver insult.

In both kidney and liver homogenates, differences in activity among the different age groups were observed toward each isosequential ASO. In the kidney, PO degradation appeared quicker in the youngest age groups than in the adult, whereas for the liver, slower degradation was observed at PND 1. Although PO are metabolized mainly by 3′-exonucleases in tissues [71,72,114,115], the activity profile for both tissues does not correlate with any of the three 3′-exonuclease expression patterns. Furthermore, no difference was seen in the degradation of the all-PS sequence across all the age groups. PS degradation appears to be more variable and cell-type specific, with either having 3′- or 5′-degradation profiles, with endonuclease-mediated degradation not generally observed [69,71,77,78]. Regarding RTR5001 degradation, the highest metabolism in the kidney was seen in the adult group, and the lowest was seen after birth until PND 3. This observation fits the expression profile of both endonucleases (*DNASE1* and *DNASE2*) and one of the 3′-exonucleases (*TREX1*).

Similarly, the nuclease activity in the liver fits the liver endonuclease (*DNASE1* and *DNASE2*) expression pattern, for which the highest activity was seen in the GD 108 group and the lowest activity was seen in the weaning and adult groups. However, this does not fit with any of the 3′-exonucleases’ expression patterns. As ASO gapmers are protected at the 3′- and 5′-end from exonuclease degradation by the additional flank modifications, they are known to be initially cleaved by endonucleases in the tissue [69,70,116] of different species [16,50], leading to short fragments that exonucleases may degrade further. As such, this supports the correlation between liver nuclease activity and endonuclease expression pattern, indicating that the nuclease activity in the liver of minipigs initially proceeds through endonucleases. As this correlation was not observed for the kidney, the nuclease activity seen in the kidney seems to be mainly due to 3′-exonucleolytic degradation and not by initial cleavage by endonucleases. Accordingly, it was observed after LNA gapmer administration in rats that only the parent compound and a metabolite with one nucleotide cleaved at the 3′-end were detected in the kidney [117]. In contrast, further degradation from the 3′-end to shorter metabolites was observed in the liver. These findings support our hypothesis regarding the metabolism of LNA gapmer in the kidney discussed above and the higher kidney-liver exposure ratio seen previously in adult minipigs [31], as metabolites with only one cleaved nucleotide cross-react more than shorter metabolites in an hELISA [108]. Hence, the differential abundance and activity of endo- and exonucleases in different organs and species can affect the metabolic pathway of LNA gapmers. Moreover, as a disparity between in vitro incubation and animal models can be observed [69,118], careful interpretation of these data, together with metabolite profiling, is warranted.

ASO degradation differed between the two organs in juvenile minipigs; i.e., unmodified ASOs were degraded faster in the kidney, whereas the LNA gapmer was degraded faster in the liver. As ASO metabolism serves to be the critical driver for its tissue elimination [101,119], the different nuclease activity in both organs and juvenile age groups relative to the adult poses a concern of potential toxicity and underdosing. However, our observation regarding the nuclease gene expression and activity is not enough to explain the exposure profile seen in the different age groups of minipigs. Other factors, such as plasma and cell-surface protein binding, extracellular matrix binding, tissue saturation, and organ maturity, should be considered and further investigated [76,101,104,120,121,122].

In general, the juvenile minipigs showed a similar toxicity profile after RTR5001 administration as in the adult minipig [31]. Clinical observations, clinical pathology, gross, and histopathological findings were similar to those observed in adult minipigs. In addition to renal tubular degeneration/regeneration, glomerulosclerosis was observed in the piglets at PND 43 and 51, which was considered to be due to repeated dosing of the test compound. Accumulation of LNA ASO in the lymph nodes as evidenced by IHC and ISH was observed, which was similar to that seen in the adult minipig and other species [13,31,112]. Likewise, no clinical or gross post-mortem observations were noted aside from the expected inflammatory reaction regarding the injection site, as in adult minipigs [31].

There was no apparent decrease in the total complement after each RTR5001 administration compared to the pre-dose levels, which corresponds to previous findings in adults of other species and humans [123,124]. This is also in accordance with the findings in the adult minipig, suggesting a lower sensitivity of minipigs for this parameter than NHPs, which are known to be over-predictive for man [31,125,126]. The total complement level was not detectable at PND 1, but the adult (control) level [31] was approximately reached from PND 8 onwards. This observation is in line with what has been seen in newborn humans [127]. On the other hand, the increase in total complement level measured pre-dose at PND 29 until 51 after repeated administration of LNA ASO could be due to chronic inflammation [127]. ASOs exhibit pro-inflammatory characteristics [13,29,128], which are seen in the juvenile minipigs as inflammatory changes in the kidneys, the accumulation of mononuclear cells in the lymph nodes, and chronic inflammation with fibrosis at the injection sites.

Regarding pharmacologic action, binding to the RNA target is critical for the activity of ASOs [129]. Moreover, the nonclinical safety package of ASOs should include a pharmacologically cross-reactive species. Therefore, the selected species should express a homologous RNA target. In this respect, the Göttingen Minipig is a potential alternative non-rodent model due to its phylogenetic proximity with humans, and this is made possible by its recent genome annotation. Despite the fact that RTR5001 has a single-end mismatch to the minipig RNA target, its location in the sequence does not ablate its pharmacologic activity [31]. The pharmacologic activity of RTR5001, i.e., decreasing total cholesterol and LDL cholesterol, was similar in the juvenile and adult minipig, but only from PND 43 onwards when compared to the controls and previous published data [62]. The higher total cholesterol values observed for the treated groups at PND 29 and 37 are within the range of historical control data for minipigs [62]. However, the total cholesterol values seen for the treated PND 23 piglets are higher than the available PND 28 data in minipigs. As these published cholesterol values were observed to increase gradually from PND 2 and peak at PND 28, we hypothesize that the actual peak is somewhere in between PND 21 and 28, requiring further investigation to address this observation.

The late onset of pharmacologic effect in the juvenile minipig could be due to several factors. Particular cell-type-specific nucleic acid-binding surface proteins [93,94,121] have been identified and were noted to be essential for cellular uptake. The presence and abundance of these proteins can affect the tissue disposition of ASOs [76,130,131]. Moreover, once inside the cell, ASOs could bind to specific proteins and get sequestered away from the target RNA and RNase H [121,132,133,134,135]. More broadly, aside from binding to ASOs inside the cell, some proteins also contribute to their activity [22,136]. Therefore, we hypothesize that the specific proteins involved in the uptake and trafficking [137] of ASO are also under-expressed in the neonate. Thus, further investigation is needed on the ontogeny of these proteins. Moreover, other factors such as the turn-over kinetics of protein translation [101,138,139] and rate of ASO-directed RNase H activity (usually minutes to hours) [121] can have an impact on the degradation of the target RNA. As such, these factors could influence the dose to be used.

In addition, the abundance of RNase H1 is another critical component that can affect the ASO-driven cleavage mechanism [121]. Both *RNASEH1* and RN*ASEH2* were included in our panel of genes for expression analysis, even though RNase H1 is considered responsible for RNA degradation in the RNA–DNA heteroduplex [52] in both the cytoplasm and nucleus [140]. On the other hand, RNase H2 has been reported to be mainly localized in the nucleus and associated with chromatin, which would impede its participation in the antisense effects of ASOs [8]. However, the 15PC3 cell line presents RNase H2 in both the cytoplasm and nucleus instead of the strict nuclear localization in other cell lines [53]. This suggests that depending on cell type, the subcellular localization of RNase H2 can have catalytic activity toward the RNA in duplex. As we were supposed to do an in vitro RNase H assay using tissue homogenates, seeing the link between RNase H1 and H2 expression with degradation of ASO substrate would be necessary, since RNase H2 is fully capable of degrading ASO in cell lysates [8,53]. However, no activity was seen in vitro (data not shown) with our biobank samples. This could be due to the low abundance of both enzymes or unspecific reaction binding in the homogenate itself from other proteins [121,132,133,134,135].

The expression of *RNASEH1* showed a different ontogeny profile for kidney and liver, with its expression level gradually increasing in the kidney and decreasing until weaning in the liver followed by an increased again toward adulthood for females. The *PCSK9*, the RNA targeted by RTR5001, is found abundantly in the liver and was successfully knocked down previously by LNA ASOs in the adult minipig, human, and several other species [26,31,47,141,142]. The higher expression level of *RNASEH1* observed in the liver of adult females than the other juvenile age–sex groups suggests that the RNase H1 level may play a role in the delayed pharmacologic effect seen in the juvenile minipigs, since the data on adults were only from females.

### Study Limitations

In our in vivo study, we were limited in sample size for the treated and the control animals, which could cause sample bias and reduced power. Therefore, we recommend cautious interpretation and extrapolation of these results. Despite the small group size, we still observed differences in ASO exposure and pharmacodynamic activity in the different age groups of Göttingen Minipigs, which was one of the goals of our study. For the ontogeny analysis, the inclusion of biobank samples was needed to increase the power of our study. This clearly shows that the correct sample size in a study is dependent on the (variability of the) endpoints that are investigated.

## 5. Conclusions

In conclusion, a similar toxicity profile was noted in juvenile minipigs as previously reported in adult minipigs following repeated ASO administration. Lower plasma and tissue exposure to RTR5001 were noted in younger minipigs up to weaning than in older or adult minipigs. Differences in the pharmacodynamic profile were also noted between minipigs of various ages. These differences in exposure and pharmacologic activity were partly explained by our nuclease ontogeny data, indicating that the juvenile Göttingen Minipig is a promising nonclinical model for the pediatric safety assessment of ASOs. Although we have to acknowledge the limited number of animals used in the in vivo study, our results highlight the importance of considering maturational factors in ASO dose setting in the pediatric population.

## Figures and Tables

**Table 2:** Clinical pathology evaluation overview. Comparison with published adult data [31] after RTR5001 administration.

Parameters	Age								
	PND 2 (*n* = 2)	PND 9 (*n* = 2)	PND 16 (*n* = 1)	PND 23 (*n* = 3)	PND 29 (*n* = 2)	PND 37 (*n* = 2)	PND 43 (*n* = 1)	PND 51 (*n* = 2)	Adult (4–6 mos) (*n* = 3)
Hematology				Minimal to mild ↑ WBC, lymphocytes		Minimal to mild ↑ WBC, neutrophils, lymphocytes	Minimal to mild ↑ WBC, neutrophils		
Clinical chemistry	Mild ↑ AST			Minimal to mild ↑ fibrinogen	Mild ↑ AST	Minimal to mild ↑ fibrinogen	Mild ↑ AST; minimal ↓ CHOL, LDL, minimal to mild ↑ fibrinogen	Minimal ↓ CHOL, LDL	↓ CHOL, LDL, trig; ↑ crea in 1 of 3, BUN
Urinalysis									↑ Na/Crea, Ca/Crea
Total complement activity	Not detectable				↑ CH50	↑ CH50	↑ CH50	↑ CH50	No data

Abbreviations: AST, aspartate aminotransferases; BUN, blood urea nitrogen; Ca, calcium; CH50, 50% hemolytic complement activity; CHOL, total cholesterol; Crea, creatinine; LDL, low-density lipoprotein; Na, sodium; PND, postnatal day; trig, triglycerides; WBC, white blood cells.

## Supplementary Materials

The following are available online at https://www.mdpi.com/article/10.3390/pharmaceutics13091442/s1, Figure S1: Mean ± SD plasma concentration after subcutaneous administration of RTR5001 to juvenile minipigs at 20 mg/kg dose level. Figure S2: Immunohistochemistry and in situ hybridization. Figure S3: Relative gene expression of nucleases for ASO metabolism and pharmacologic activity between kidney and liver over time in postnatal and adult Göttingen Minipig. Figure S4: Relative gene expression of nucleases for ASO metabolism and pharmacologic activity over time in the developing and adult Göttingen Minipig samples from the investigational toxicity study. Figure S5: Estimated reaction rate toward the isosequential ASOs: unmodified (PO), all-PS (PS), and LNA/PS/LNA gapmer in the kidney and liver homogenates from the developing and adult Göttingen Minipig. Figure S6: Published mean ± SD exposure levels in the plasma; mean ± SD maximum plasma concentration (Cmax); and mean exposure levels in the kidney and liver data of adult Göttingen Minipig after RTR5001 administration.

## References

[1] Dias N., Stein C.A. (2002). Antisense oligonucleotides: Basic concepts and mechanisms. Mol. Cancer Ther..

[2] Mustonen E.K., Palomäki T., Pasanen M. (2017). Oligonucleotide-based pharmaceuticals: Non-clinical and clinical safety signals and non-clinical testing strategies. Regul. Toxicol. Pharmacol..

[3] Xiong H., Veedu R.N., Diermeier S.D. (2021). Recent advances in oligonucleotide therapeutics in oncology. Int. J. Mol. Sci..

[4] Quemener A.M., Bachelot L., Forestier A., Donnou-Fournet E., Gilot D., Galibert M.D. (2020). The powerful world of antisense oligonucleotides: From bench to bedside. Wiley Interdiscip. Rev. RNA.

[5] Crooke S.T. (2017). Molecular Mechanisms of Antisense Oligonucleotides. Nucleic Acid Ther..

[6] Dhuri K., Bechtold C., Quijano E., Pham H., Gupta A., Vikram A., Bahal R. (2020). Antisense Oligonucleotides: An Emerging Area in Drug Discovery and Development. J. Clin. Med..

[7] Yin W., Rogge M. (2019). Targeting RNA: A Transformative Therapeutic Strategy. Clin. Transl. Sci..

[8] Lima W., Wu H., Crooke S.T., Crooke S.T. (2007). The RNase H mechanism. Antisense Drug Technology: Principles, Strategies, and Applications.

[9] Henry S.P., Johnson M., Zanardi T.A., Fey R., Auyeung D., Lappin P.B., Levin A.A. (2012). Renal uptake and tolerability of a 2’-O-methoxyethyl modified antisense oligonucleotide (ISIS 113715) in monkey. Toxicology.

[10] Brown D.A., Kang S.H., Gryaznov S.M., DeDionisio L., Heidenreich O., Sullivan S., Xu X., Nerenberg M.I. (1994). Effect of phosphorothioate modification of oligodeoxynucleotides on specific protein binding. J. Biol. Chem..

[11] Geary R.S., Watanabe T.A., Truong L., Freier S., Lesnik E.A., Sioufi N.B., Sasmor H., Manoharan M., Levin A.A. (2001). Pharmacokinetic properties of 2′-O-(2-methoxyethyl)-modified oligonucleotide analogs in rats. J. Pharmacol. Exp. Ther..

[12] Geary R.S., Norris D., Yu R., Bennett C.F. (2015). Pharmacokinetics, biodistribution and cell uptake of antisense oligonucleotides. Adv. Drug Deliv. Rev..

[13] Frazier K.S. (2015). Antisense Oligonucleotide Therapies:The Promise and the Challenges from a Toxicologic Pathologist’s Perspective. Toxicol. Pathol..

[14] Yu R.Z., Geary R.S., Flaim J.D., Riley G.C., Tribble D.L., VanVliet A.A., Wedel M.K. (2009). Lack of pharmacokinetic interaction of mipomersen sodium (ISIS 301012), a 2′-O-methoxyethyl modified antisense oligonucleotide targeting apolipoprotein B-100 messenger RNA, with simvastatin and ezetimibe. Clin. Pharmacokinet..

[15] Kazmi F., Yerino P., McCoy C., Parkinson A., Buckley D.B., Ogilvie B.W. (2018). An assessment of the in vitro inhibition of cytochrome P450 enzymes, UDP-glucuronosyltransferases, and transporters by phosphodiester- or phosphorothioate-linked oligonucleotides. Drug Metab. Dispos..

[16] Yu R.Z., Kim T.W., Hong A., Watanabe T.A., Gaus H.J., Geary R.S. (2007). Cross-species pharmacokinetic comparison from mouse to man of a second-generation antisense oligonucleotide, ISIS 301012, targeting human apolipoprotein B-100. Drug Metab. Dispos..

[17] Griffey R.H., Greig M.J., Gaus H.J., Liu K., Monteith D., Winniman M., Cummins L.L. (1997). Characterization of oligonucleotide metabolism in vivo via liquid chromatography/electrospray tandem mass spectrometry with a quadrupole ion trap mass spectrometer. J. Mass Spectrom..

[18] Yang W. (2011). Nucleases: Diversity of structure, function and mechanism. Q. Rev. Biophys..

[19] Eder P.S., Devine R.J., Dagle J.M., Walder J.A. (1991). Substrate Specificity and Kinetics of Degradation of Antisense Oligonucleotides by a 3′ Exonuclease in Plasma. Antisense Res. Dev..

[20] Wójcik M., Cieślak M., Stec W.J., Goding J.W., Koziołkiewicz M. (2007). Nucleotide Pyrophosphatase/Phosphodiesterase 1 Is Responsible for Degradation of Antisense Phosphorothioate Oligonucleotides. Oligonucleotides.

[21] Geary R.S. (2009). Antisense oligonucleotide pharmacokinetics and metabolism. Expert Opin. Drug Metab. Toxicol..

[22] Watts J.K., Ferrari N., Seguin R. (2018). The Medicinal Chemistry of Antisense Oligonucleotides. Oligonucleotide-Based Drugs and Therapeutics.

[23] Krishnan A.V., Mishra D. (2020). Antisense Oligonucleotides: A Unique Treatment Approach. Indian Pediatr..

[24] Laxton C., Brady K., Moschos S., Turnpenny P., Rawal J., Pryde D.C., Sidders B., Corbau R., Pickford C., Murray E.J. (2011). Selection, optimization, and pharmacokinetic properties of a novel, potent antiviral locked nucleic acid-based antisense oligomer targeting hepatitis C virus internal ribosome entry site. Antimicrob. Agents Chemother..

[25] Kuespert S., Heydn R., Peters S., Wirkert E., Meyer A.L., Siebörger M., Johannesen S., Aigner L., Bogdahn U., Bruun T.H. (2020). Antisense oligonucleotide in LNA-gapmer design targeting TGFBR2—A key single gene target for safe and effective inhibition of TGFβ signaling. Int. J. Mol. Sci..

[26] Gupta N., Fisker N., Asselin M.C., Lindholm M., Rosenbohm C., Ørum H., Elmén J., Seidah N.G., Straarup E.M. (2010). A locked nucleic acid antisense oligonucleotide (LNA) silences PCSK9 and enhances LDLR expression In Vitro and In Vivo. PLoS ONE.

[27] Abewe H., Deshmukh S., Mukim A., Beliakova-Bethell N. (2020). Use of GapmeRs for gene expression knockdowns in human primary resting CD4+ T cells. J. Immunol. Methods.

[28] Roberts T.C., Langer R., Wood M.J.A. (2020). Advances in oligonucleotide drug delivery. Nat. Rev. Drug Discov..

[29] Hammond S.M., Aartsma-Rus A., Alves S., Borgos S.E., Buijsen R.A.M., Collin R.W.J., Covello G., Denti M.A., Desviat L.R., Echevarría L. (2021). Delivery of oligonucleotide-based therapeutics: Challenges and opportunities. EMBO Mol. Med..

[30] European Medicine Agency, Committee for Medicinal Products for Human Use, International Council for Harmonisation of Technical Requirements for Pharmaceuticas for Human Use (2013). ICH Guideline M3 (R2) on Non-Clinical Safety Studies for the Conduct of Human Clinical Trials and Marketing Authorization for Pharmaceuticals, Step 5, EMA/CHMP/ICH/286/95.

[31] Braendli-Baiocco A., Festag M., Erichsen K.D., Persson R., Mihatsch M.J., Fisker N., Funk J., Mohr S., Constien R., Ploix C. (2017). The minipig is a suitable non-rodent model in the safety assessment of single stranded oligonucleotides. Toxicol. Sci..

[32] Heckel T., Schmucki R., Berrera M., Ringshandl S., Badi L., Steiner G., Ravon M., Küng E., Kuhn B., Kratochwil N.A. (2015). Functional analysis and transcriptional output of the Göttingen minipig genome. BMC Genomics.

[33] Vamathevan J.J., Hall M.D., Hasan S., Woollard P.M., Xu M., Yang Y., Li X., Wang X., Kenny S., Brown J.R. (2013). Minipig and beagle animal model genomes aid species selection in pharmaceutical discovery and development. Toxicol. Appl. Pharmacol..

[34] Scoto M., Finkel R., Mercuri E., Muntoni F. (2018). Genetic therapies for inherited neuromuscular disorders. Lancet Child Adolesc. Heal..

[35] Abreu N.J., Waldrop M.A. (2021). Overview of gene therapy in spinal muscular atrophy and Duchenne muscular dystrophy. Pediatr. Pulmonol..

[36] Aoki Y., Wood M.J.A. (2021). Emerging Oligonucleotide Therapeutics for Rare Neuromuscular Diseases. J. Neuromuscul. Dis..

[37] Osredkar D., Jílková M., Butenko T., Loboda T., Golli T., Fuchsová P., Rohlenová M., Haberlova J. (2021). Children and young adults with spinal muscular atrophy treated with nusinersen. Eur. J. Paediatr. Neurol..

[38] Hoffman E.P. (2020). Pharmacotherapy of duchenne muscular dystrophy. Handb. Exp. Pharmacol..

[39] Martinovich K.M., Shaw N.C., Kicic A., Schultz A., Fletcher S., Wilton S.D., Stick S.M. (2018). The potential of antisense oligonucleotide therapies for inherited childhood lung diseases. Mol. Cell. Pediatr..

[40] Ashrafi M.R., Amanat M., Garshasbi M., Kameli R., Nilipour Y., Heidari M., Rezaei Z., Tavasoli A.R. (2020). An update on clinical, pathological, diagnostic, and therapeutic perspectives of childhood leukodystrophies. Expert Rev. Neurother..

[41] Oren Y.S., Irony-Tur Sinai M., Golec A., Barchad-Avitzur O., Mutyam V., Li Y., Hong J., Ozeri-Galai E., Hatton A., Leibson C. (2021). Antisense oligonucleotide-based drug development for Cystic Fibrosis patients carrying the 3849+10 kb C-to-T splicing mutation. J. Cyst. Fibros..

[42] European Medicine Agency, Committee for Medicinal Products for Human Use, International Council for Harmonisation of Technical Requirements for Pharmaceuticas for Human Use (2020). ICH Guideline S11 on Noncliniclinical Safety Testing in Support of Development of Pediatric Pharmaceuticals, Step 5, EMA/CHMP/ICH/616110/2018.

[43] Barrow P.C., Barbellion S., Stadler J. (2011). Preclinical evaluation of juvenile toxicity. Methods Mol. Biol..

[44] Ayuso M., Buyssens L., Stroe M., Valenzuela A., Allegaert K., Smits A., Annaert P., Mulder A., Carpentier S., Van Ginneken C. (2021). The neonatal and juvenile pig in pediatric drug discovery and development. Pharmaceutics.

[45] Bode G., Clausing P., Gervais F., Loegsted J., Luft J., Nogues V., Sims J. (2010). The utility of the minipig as an animal model in regulatory toxicology. J. Pharmacol. Toxicol. Methods.

[46] Forster R., Bode G., Ellegaard L., van der Laan J.W. (2010). The RETHINK project on minipigs in the toxicity testing of new medicines and chemicals: Conclusions and recommendations. J. Pharmacol. Toxicol. Methods.

[47] Lindholm M.W., Elmén J., Fisker N., Hansen H.F., Persson R., Møller M.R., Rosenbohm C., Ørum H., Straarup E.M., Koch T. (2012). PCSK9 LNA antisense oligonucleotides induce sustained reduction of LDL cholesterol in nonhuman primates. Mol. Ther..

[48] Lin X.L., Xiao L.L., Tang Z.H., Jiang Z.S., Liu M.H. (2018). Role of PCSK9 in lipid metabolism and atherosclerosis. Biomed. Pharmacother..

[49] Van Peer E., Downes N., Casteleyn C., Van Ginneken C., Weeren A., Van Cruchten S. (2016). Organ data from the developing Göttingen minipig: First steps towards a juvenile PBPK model. J. Pharmacokinet. Pharmacodyn..

[50] Geary R.S., Yu R.Z., Watanabe T., Henry S.P., Hardee G.E., Chappell A., Matson J., Sasmor H., Cummins L., Levin A.A. (2003). Pharmacokinetics of a tumor necrosis factor-α phosphorothioate 2′-O-(2-methoxyethyl) modified antisense oligonucleotide: Comparison across species. Drug Metab. Dispos..

[51] Stein H., Hausen P. (1969). Enzyme from calf thymus degrading the RNA moiety of DNA-RNA hybrids: Effect on DNA-dependent RNA polymerase. Science.

[52] Wu H., Lima W.F., Zhang H., Fan A., Sun H., Crooke S.T. (2004). Determination of the Role of the Human RNase H1 in the Pharmacology of DNA-like Antisense Drugs. J. Biol. Chem..

[53] Ten Asbroek A.L.M.A., Van Groenigen M., Nooij M., Baas F. (2002). The involvement of human ribonucleases H1 and H2 in the variation of response of cells to antisense phosphorothioate oligonucleotides. Eur. J. Biochem..

[54] Nygard A.B., Jørgensen C.B., Cirera S., Fredholm M. (2007). Selection of reference genes for gene expression studies in pig tissues using SYBR green qPCR. BMC Mol. Biol..

[55] Vandesompele J., De Preter K., Pattyn F., Poppe B., Van Roy N., De Paepe A., Speleman F. (2002). Accurate normalization of real-time quantitative RT-PCR data by geometric averaging of multiple internal control genes. Genome Biol..

[56] Wahlestedt C., Salmi P., Good L., Kela J., Johnsson T., Ho T., Broberger C., Porreca F., Lai J., Ren K. (2000). Potent and nontoxic antisense oligonucleotides. Proc. Natl. Acad. Sci. USA..

[57] Gilar M., Belenky A., Smisek D.L., Bourque A., Cohen A.S. (1997). Kinetics of phosphorothioate oligonucleotide metabolism in biological fluids. Nucleic Acids Res..

[58] Mazur D.J., Perrino F.W. (1999). Identification and expression of the TREX1 and TREX2 cDNA sequences encoding mammalian 3′→5′ exonucleases. J. Biol. Chem..

[59] Kavanagh D., Spitzer D., Kothari P.H., Shaikh A., Liszewski M.K., Richards A., Atkinson J.P. (2010). New roles for the major human 3′–5′ exonuclease TREX1 in human disease. Cell Cycle.

[60] Kishi K., Yasuda T., Ikehara Y., Sawazaki K., Sato W., Ida R. (1990). Human serum deoxyribonuclease I (DNase I) polymorphism: Pattern similarities among isozymes from serum, urine, kidney, liver, and pancreas. Am. J. Hum. Genet..

[61] Evans C.J., Aguilera R.J. (2003). DNase II: Genes, enzymes and function. Gene.

[62] Grossi A.B., Zeltner A., Christoffersen C., Søndergaard A.C. (2016). Reference data of clinical chemistry and hematology in juvenile Göttingen Minipigs. Toxicol. Lett..

[63] Seely J.C. (2017). A brief review of kidney development, maturation, developmental abnormalities, and drug toxicity: Juvenile animal relevancy. J. Toxicol. Pathol..

[64] Szudzik M., Starzyński R.R., Jończy A., Mazgaj R., Lenartowicz M., Lipiński P. (2019). Erratum: Iron supplementation in suckling piglets: An ostensibly easy therapy of neonatal iron deficiency anemia. Pharmaceuticals.

[65] O’Hara K. (2016). Paediatric pharmacokinetics and drug doses. Aust. Prescr..

[66] Van Donge T., Evers K., Koch G., van den Anker J., Pfister M. (2020). Clinical pharmacology and pharmacometrics to better understand physiological changes during pregnancy and neonatal life. Handb. Exp. Pharmacol..

[67] Bueters R., Bael A., Gasthuys E., Chen C., Schreuder M.F., Frazier K.S. (2020). Ontogeny and Cross-species Comparison of Pathways Involved in Drug Absorption, Distribution, Metabolism, and Excretion in Neonates (Review): Kidney. Drug Metab. Dispos..

[68] Kurreck J., Wyszko E., Gillen C., Erdmann V.A. (2002). Design of antisense oligonucleotides stabilized by locked nucleic acids. Nucleic Acids Res..

[69] Kim J., Basiri B., Hassan C., Punt C., van der Hage E., den Besten C., Bartlett M.G. (2019). Metabolite Profiling of the Antisense Oligonucleotide Eluforsen Using Liquid Chromatography-Mass Spectrometry. Mol. Ther. Nucleic Acid.

[70] Baek M., Yu R.Z., Gaus H., Grundy J.S., Geary R.S. (2010). In Vitro Metabolic Stabilities and Metabolism Antisense Oligonucleotides in Preincubated Rat or Human Whole Liver Homogenates. Oligonucleotides.

[71] Crooke R.M., Graham M.J., Martin M.J., Lemonidis K.M., Wyrzykiewiecz T.A.D., Cummins L.L. (2000). Metabolism of Antisense Oligonucleotides in Rat Liver Homogenates. J. Pharmacol. Exp. Ther..

[72] Sands H., Gorey-Feret L.J., Cocuzza A.J., Hobbs F.W., Chidester D., Trainor G.L. (1994). Biodistribution and metabolism of internally 3H-labeled oligonucleotides. I. Comparison of a phosphodiester and a phosphorothioate. Mol. Pharmacol..

[73] Walder R.Y., Walder J.A. (1988). Role of RNase H in hybrid-arrested translation by antisense oligonucleotides. Proc. Natl. Acad. Sci. USA.

[74] Lin M., Hu X., Chang S., Chang Y., Bian W., Hu R., Wang J., Zhu Q., Qiu J. (2021). Advances of Antisense Oligonucleotide Technology in the Treatment of Hereditary Neurodegenerative Diseases. Evidence-based Complement. Altern. Med..

[75] Chen S., Le B.T., Chakravarthy M., Kosbar T.R., Veedu R.N. (2019). Systematic evaluation of 2′-Fluoro modified chimeric antisense oligonucleotide-mediated exon skipping in vitro. Sci. Rep..

[76] Dirin M., Winkler J., Ferrari N., Seguin R. (2018). Tissue Distribution, Metabolism, and Clearance. Oligonucleotide-Based Drugs and Therapeutics.

[77] Levin A.A. (1999). A review of issues in the pharmacokinetics and toxicology of phosphorothioate antisense oligonucleotides. Biochim. Biophys. Acta.

[78] Gaus H.J., Owens S.R., Winniman M., Cooper S., Cummins L.L. (1997). On-Line HPLC Electrospray Mass Spectrometry of Phosphorothioate Oligonucleotide Metabolites. Anal. Chem..

[79] Shaw J., Kent K., Bird J., Fishback J., Froehler B. (1991). Modified deoxyoligonucleotides stable to exonuclease degradation in serum. Nucleic Acids Res..

[80] Østergaard M.E., De Hoyos C.L., Wan W.B., Shen W., Low A., Berdeja A., Vasquez G., Murray S., Migawa M.T., Liang X.H. (2020). Understanding the effect of controlling phosphorothioate chirality in the DNA gap on the potency and safety of gapmer antisense oligonucleotides. Nucleic Acids Res..

[81] Belli S.I., van Driel I.R., Goding J.W. (1993). Identification and characterization of a soluble form of the plasma cell membrane glycoprotein PC-1 (5′-nucleotide phosphodiesterase). Eur. J. Biochem..

[82] Loughney K., Ferguson K., Schudt C., Dent G., Rabe K. (1996). Identification and Quantification of PDE Isoenzymes and Subtypes by Molecular Biological Methods. Phosphodiesterase Inhibitors.

[83] Wang D. (2008). Discrepancy between mRNA and protein abundance: Insight from information retrieval process in computers. Comput. Biol. Chem..

[84] Bruckmueller H., Martin P., Kähler M., Haenisch S., Ostrowski M., Drozdzik M., Siegmund W., Cascorbi I., Oswald S. (2017). Clinically Relevant Multidrug Transporters Are Regulated by microRNAs along the Human Intestine. Mol. Pharm..

[85] Zapalska-Sozoniuk M., Chrobak L., Kowalczyk K., Kankofer M. (2019). Is it useful to use several “omics” for obtaining valuable results?. Mol. Biol. Rep..

[86] Kamaliddin C., Guillochon E., Salnot V., Rombaut D., Huguet S., Guillonneau F., Houzé S., Cot M., Deloron P., Argy N. (2021). Comprehensive Analysis of Transcript and Protein Relative Abundance during Blood Stages of Plasmodium falciparum Infection. J. Proteome Res..

[87] Yu R.Z., Geary R.S., Levin A.A., Meibohm B. (2006). Pharmacokinetics and Pharmacodynamics of Antisense Oligonucleotides. Pharmacokinetics and Pharmacodynamics of Biotech Drugs: Principles and Case Studies in Drug Development.

[88] Lundin K.E., Hansen B.R., Persson R., Bramsen J.B., Koch T., Wengel J., Smith C.I.E. (2013). Biological Activity and Biotechnological Aspects of Locked Nucleic Acids. Adv. Genet..

[89] Post N., Yu R., Greenlee S., Gaus H., Hurh E., Matson J., Wang Y. (2019). Metabolism and disposition of volanesorsen, a 29-O-(2 methoxyethyl) antisense oligonucleotide, across species. Drug Metab. Dispos..

[90] Tillman L.G., Geary R.S., Hardee G.E. (2008). Oral delivery of antisense oligonucleotides in man. J. Pharm. Sci..

[91] Crooke S.T., Geary R.S. (2013). Clinical pharmacological properties of mipomersen ( Kynamro ), a second generation antisense inhibitor of apolipoprotein B. Br. J. Clin. Pharmacol..

[92] Yu R.Z., Grundy J.S., Geary R.S. (2013). Clinical pharmacokinetics of second generation antisense oligonucleotides. Expert Opin. Drug Metab. Toxicol..

[93] Schmidt K., Prakash T.P., Donner A.J., Kinberger G.A., Gaus H.J., Low A., Østergaard M.E., Bell M., Swayze E.E., Seth P.P. (2017). Characterizing the effect of GalNAc and phosphorothioate backbone on binding of antisense oligonucleotides to the asialoglycoprotein receptor. Nucleic Acids Res..

[94] Miller C.M., Donner A.J., Blank E.E., Egger A.W., Kellar B.M., Østergaard M.E., Seth P.P., Harris E.N. (2016). Stabilin-1 and Stabilin-2 are specific receptors for the cellular internalization of phosphorothioate-modified antisense oligonucleotides (ASOs) in the liver. Nucleic Acids Res..

[95] Hvam M.L., Cai Y., Dagnæs-Hansen F., Nielsen J.S., Wengel J., Kjems J., Howard K.A. (2017). Fatty Acid-Modified Gapmer Antisense Oligonucleotide and Serum Albumin Constructs for Pharmacokinetic Modulation. Mol. Ther..

[96] Migawa M.T., Shen W., Wan W.B., Vasquez G., Oestergaard M.E., Low A., De Hoyos C.L., Gupta R., Murray S., Tanowitz M. (2019). Site-specific replacement of phosphorothioate with alkyl phosphonate linkages enhances the therapeutic profile of gapmer ASOs by modulating interactions with cellular proteins. Nucleic Acids Res..

[97] Srinivasan S.K., Tewary H.K., Iversen P.L. (1995). Characterization of Binding Sites, Extent of Binding, and Drug Interactions of Oligonucleotides with Albumin. Antisense Res. Dev..

[98] Watanabe T.A., Geary R.S., Levin A.A. (2006). Plasma protein binding of an antisense oligonucleotide targeting human ICAM-1 (ISIS 2302). Oligonucleotides.

[99] Wartlick H., Spänkuch-Schmitt B., Strebhardt K., Kreuter J., Langer K. (2004). Tumour cell delivery of antisense oligonuclceotides by human serum albumin nanoparticles. J. Control. Release.

[100] Gaus H.J., Gupta R., Chappell A.E., Østergaard M.E., Swayze E.E., Seth P.P. (2019). Characterization of the interactions of chemically-modified therapeutic nucleic acids with plasma proteins using a fluorescence polarization assay. Nucleic Acids Res..

[101] Lightfoot H., Schneider A., Hall J., Ferrari N., Seguin R. (2018). Pharmacokinetics and Pharmacodynamics of Antisense Oligonucleotides. Oligonucleotide-Based Drugs and Therapeutics.

[102] Lanford R.E., Hildebrandt-Eriksen E.S., Petri A., Persson R., Lindow M., Munk M.E., Kauppinen S., Rum H. (2010). Therapeutic silencing of microRNA-122 in primates with chronic hepatitis C virus infection. Science..

[103] Weidolf L., Björkbom A., Dahlén A., Elebring M., Gennemark P., Hölttä M., Janzén D., Li X.Q., Andersson S. (2021). Distribution and biotransformation of therapeutic antisense oligonucleotides and conjugates. Drug Discov. Today.

[104] Goemans N.M., Tulinius M., van den Akker J.T., Burm B.E., Ekhart P.F., Heuvelmans N., Holling T., Janson A.A., Platenburg G.J., Sipkens J.A. (2011). Systemic Administration of PRO051 in Duchenne’s Muscular Dystrophy. N. Engl. J. Med..

[105] Levin A.A., Yu R.Z., Geary R.S., Crooke S.T. (2007). Basic principles of the pharmacokinetics of antisense oligonucleotide drugs. Antisense Drug Technology: Principles, Strategies, and Applications.

[106] Straarup E.M., Fisker N., Hedtjärn M., Lindholm M.W., Rosenbohm C., Aarup V., Hansen H.F., Ørum H., Hansen J.B.R., Koch T. (2010). Short locked nucleic acid antisense oligonucleotides potently reduce apolipoprotein B mRNA and serum cholesterol in mice and non-human primates. Nucleic Acids Res..

[107] Hagedorn P.H., Persson R., Funder E.D., Albæk N., Diemer S.L., Hansen D.J., Møller M.R., Papargyri N., Christiansen H., Hansen B.R. (2018). Locked nucleic acid: Modality, diversity, and drug discovery. Drug Discov. Today.

[108] Wei X., Dai G., Marcucci G., Liu Z., Hoyt D., Blum W., Chan K.K. (2006). A specific picomolar hybridization-based ELISA assay for the determination of phosphorothioate oligonucleotides in plasma and cellular matrices. Pharm. Res..

[109] Deverre J.R., Boutet V., Boquet D., Ezan E., Grassi J., Grognet J.M. (1997). A competitive enzyme hybridization assay for plasma determination of phosphodiester and phosphorothioate antisense oligonucleotides. Nucleic Acids Res..

[110] Andersson S., Antonsson M., Elebring M., Jansson-Löfmark R., Weidolf L. (2018). Drug metabolism and pharmacokinetic strategies for oligonucleotide- and mRNA-based drug development. Drug Discov. Today.

[111] Frazier K.S. (2017). Species Differences in Renal Development and Associated Developmental Nephrotoxicity. Birth Defects Res..

[112] Hildebrandt-Eriksen E.S., Aarup V., Persson R., Hansen H.F., Munk M.E., Ørum H. (2012). A locked nucleic acid oligonucleotide targeting microRNA 122 is well-tolerated in cynomolgus monkeys. Nucleic Acid Ther..

[113] Koch T., Ørum H., Crooke S.T. (2007). Locked nucleic acid. Antisense Drug Technology: Principles, Strategies, and Applications.

[114] Graham M.J., Crooke S.T., Monteith D.K., Cooper S.R., Lemonidis K.M., Stecker K.I.M.K., Martin M.J., Crooke R.M. (1998). In Vivo Distribution and Metabolism of a Phosphorothioate Oligonucleotide within Rat Liver after Intravenous Administration. J. Pharmacol. Exp..

[115] Altmann K.H., Dean N.M., Fabbro D., Freier S.M., Geiger T., Hanera R., Hiisken D.A., Martina P., Monia P.B., Miiller M. (1996). Second generation of antisense oligonucleotides: From nuclease resistance to biological efficacy in animals. Chim. Int. J. Chem..

[116] Kim J., El Zahar N.M., Bartlett M.G. (2020). In vitro metabolism of 2′-ribose unmodified and modified phosphorothioate oligonucleotide therapeutics using liquid chromatography mass spectrometry. Biomed. Chromatogr..

[117] Romero-Palomo F., Festag M., Lenz B., Schadt S., Brink A., Kipar A., Steinhuber B., Husser C., Koller E., Sewing S. (2021). Safety, Tissue Distribution, and Metabolism of LNA-Containing Antisense Oligonucleotides in Rats. Toxicol. Pathol..

[118] Kilanowska A., Studzińska S. (2020). In vivo and in vitro studies of antisense oligonucleotides—a review. RSC Adv..

[119] White P.J., Anastasopoulos F., Pouton C.W., Boyd B.J. (2009). Overcoming biological barriers to in vivo efficacy of antisense oligonucleotides. Expert Rev. Mol. Med..

[120] Yu R.Z., Lemonidis K.M., Graham M.J., Matson J.E., Crooke R.M., Tribble D.L., Wedel M.K., Levin A.A., Geary R.S. (2009). Cross-species comparison of in vivo PK/PD relationships for second-generation antisense oligonucleotides targeting apolipoprotein B-100. Biochem. Pharmacol..

[121] Crooke S.T., Liang X.H., Baker B.F., Crooke R.M. (2021). Antisense technology: A review. J. Biol. Chem..

[122] Geary R.S., Yu R.Z., Siwkowski A., Levin A.A., Crooke S.T. (2007). Pharmacokinetic/Pharmacodynamic Properties of Phosphorothioate 2′-O-(2-Methoxyethyl)-Modified Antisense Oligonucleotides in Animals and Man. Antisense Drug Technology: Principles, Strategies, and Applications.

[123] Henry S.P., Jagels M.A., Hugli T.E., Manalili S., Geary R.S., Giclas P.C., Levin A.A. (2014). Mechanism of alternative complement pathway dysregulation by a phosphorothioate oligonucleotide in monkey and human serum. Nucleic Acid Ther..

[124] Shen L., Engelhardt J.A., Hung G., Yee J., Kikkawa R., Matson J., Tayefeh B., Machemer T., Giclas P.C., Henry S.P. (2016). Effects of Repeated Complement Activation Associated with Chronic Treatment of Cynomolgus Monkeys with 2′-O-Methoxyethyl Modified Antisense Oligonucleotide. Nucleic Acid Ther..

[125] Henry S.P., Seguin R., Cavagnaro J., Berman C., Tepper J., Kornbrust D. (2016). Considerations for the Characterization and Interpretation of Results Related to Alternative Complement Activation in Monkeys Associated with Oligonucleotide-Based Therapeutics. Nucleic Acid Ther..

[126] Andersson P., Den Besten C., Agrawal S., Gait M. (2019). Preclinical and Clinical Drug-metabolism, Pharmacokinetics and Safety of Therapeutic Oligonucleotides. RSC Drug Discovery Series: Advances in Nucleic Acid Therapeutics.

[127] Mcgreal E.P., Hearne K., Spiller O.B. (2012). Immunobiology Off to a slow start: Under-development of the complement system in term newborns is more substantial following premature birth. Immunobiology.

[128] Seguin R., Ferrari N., Seguin R. (2018). Class-Related Proinflammatory Effects. Oligonucleotide-Based Drugs and Therapeutics.

[129] Aartsma-Rus A., Jackson A.L., Levin A.A., Ferrari N., Seguin R. (2018). Mechanisms of Oligonucleotide Actions. Oligonucleotide-Based Drugs and Therapeutics.

[130] Geary R.S., Wancewicz E., Matson J., Pearce M., Siwkowski A., Swayze E., Bennett F. (2009). Effect of dose and plasma concentration on liver uptake and pharmacologic activity of a 2′-methoxyethyl modified chimeric antisense oligonucleotide targeting PTEN. Biochem. Pharmacol..

[131] Koller E., Vincent T.M., Chappell A., De S., Manoharan M., Bennett C.F. (2011). Mechanisms of single-stranded phosphorothioate modified antisense oligonucleotide accumulation in hepatocytes. Nucleic Acids Res..

[132] Liang X.H., Sun H., Shen W., Crooke S.T. (2015). Identification and characterization of intracellular proteins that bind oligonucleotides with phosphorothioate linkages. Nucleic Acids Res..

[133] Pollak A.J., Hickman J.H., Liang X.H., Crooke S.T. (2020). Gapmer Antisense Oligonucleotides Targeting 5S Ribosomal RNA Can Reduce Mature 5S Ribosomal RNA by Two Mechanisms. Nucleic Acid Ther..

[134] Vickers T.A., Crooke S.T. (2014). Antisense oligonucleotides capable of promoting specific target mRNA reduction via competing RNase H1-dependent and independent mechanisms. PLoS ONE.

[135] Vickers T.A., Sabripour M., Crooke S.T. (2011). U1 adaptors result in reduction of multiple pre-mRNA species principally by sequestering U1snRNP. Nucleic Acids Res..

[136] Crooke S.T., Wang S., Vickers T.A., Shen W., Liang X.H. (2017). Cellular uptake and trafficking of antisense oligonucleotides. Nat. Biotechnol..

[137] Liang X.H., Sun H., Nichols J.G., Allen N., Wang S., Vickers T.A., Shen W., Hsu C.W., Crooke S.T. (2018). COPII vesicles can affect the activity of antisense oligonucleotides by facilitating the release of oligonucleotides from endocytic pathways. Nucleic Acids Res..

[138] Ramanathan M., Macgregor R.D., Hunt C.A. (1993). Predictions of Effect for Intracellular Antisense Oligodeoxyribonucleotides from a Kinetic Model. Antisense Res. Dev..

[139] Spiller D.G., Giles R.V., Broughton C.M., Grzybowski J., Ruddell C.J., Tidd D.M., Clark R.E. (1998). The influence of target protein half-life on the effectiveness of antisense oligonucleotide analog-mediated biologic responses. Antisense Nucleic Acid Drug Dev..

[140] Liang X.H., Sun H., Nichols J.G., Crooke S.T. (2017). RNase H1-Dependent Antisense Oligonucleotides Are Robustly Active in Directing RNA Cleavage in Both the Cytoplasm and the Nucleus. Mol. Ther..

[141] Van Poelgeest E.P., Swart R.M., Betjes M.G.H., Moerland M., Weening J.J., Tessier Y., Hodges M.R., Levin A.A., Burggraaf J. (2013). Acute kidney injury during therapy with an antisense oligonucleotide directed against PCSK9. Am. J. Kidney Dis..

[142] Van Poelgeest E.P., Hodges M.R., Moerland M., Tessier Y., Levin A.A., Persson R., Lindholm M.W., Dumong Erichsen K., Ørum H., Cohen A.F. (2015). Antisense-mediated reduction of proprotein convertase subtilisin/kexin type 9 (PCSK9): A first-in-human randomized, placebo-controlled trial. Br. J. Clin. Pharmacol..

