# Supplementary material for: Safety Testing of an Antisense Oligonucleotide Intended for Pediatric Indications in the Juvenile Göttingen Minipig, including an Evaluation of the Ontogeny of Key Nucleases
Source: Pharmaceutics. 2021 Sep 10;13(9):1442. doi: 10.3390/pharmaceutics13091442 (PMC8470776; doi:10.3390/pharmaceutics13091442)
Supplement: Supplementary file 1 [file pharmaceutics-13-01442-s001.zip › pharmaceutics-1336997-supplementary.pdf]

pharmaceutics

Safety Testing of an Antisense Oligonucleotide Intended for Pediatric Indications in the Juvenile Göttingen Minipig, Including an Evaluation of the Ontogeny of Key Nucleases

Allan Valenzuela, Claire Tardiveau, Miriam Ayuso, Laura Buyssens, Chloe Bars, Chris Van Ginneken, Pierluigi Fant, Isabelle Leconte, Annamaria Braendli-Baiocco, Neil Parrott, Georg Schmitt, Yann Tessier, Paul Barrow, and Steven Van Cruchten

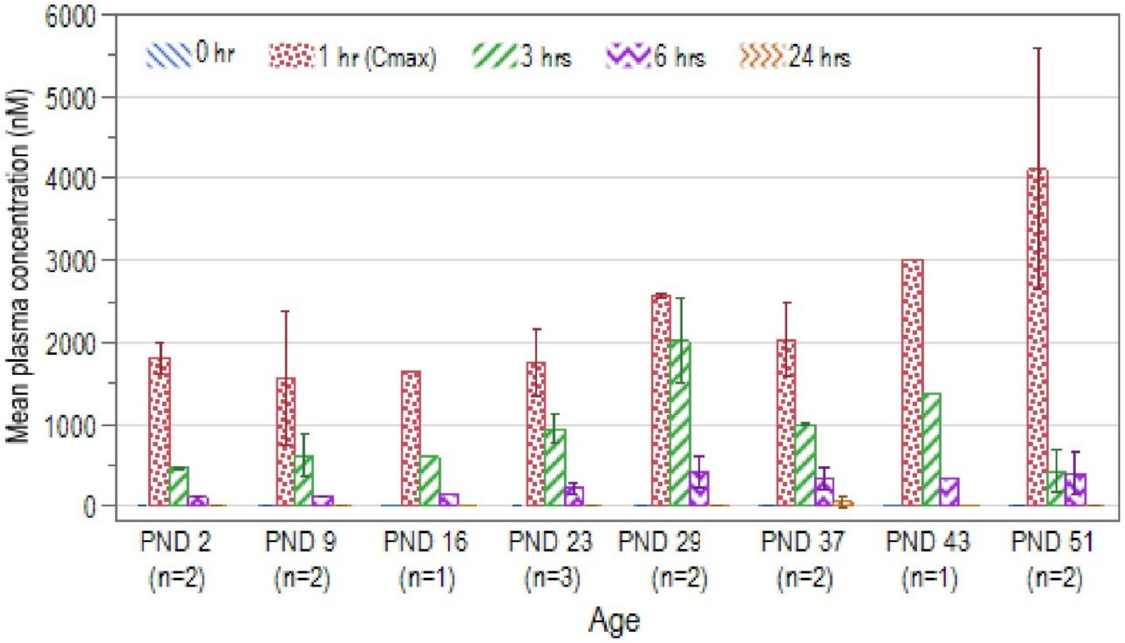

Figure S1. Mean ± SD plasma concentration after subcutaneous administration of RTR5001 to juvenile minipigs at 20mg/kg dose level. 0 h: trough level; Cmax: maximum concentration.

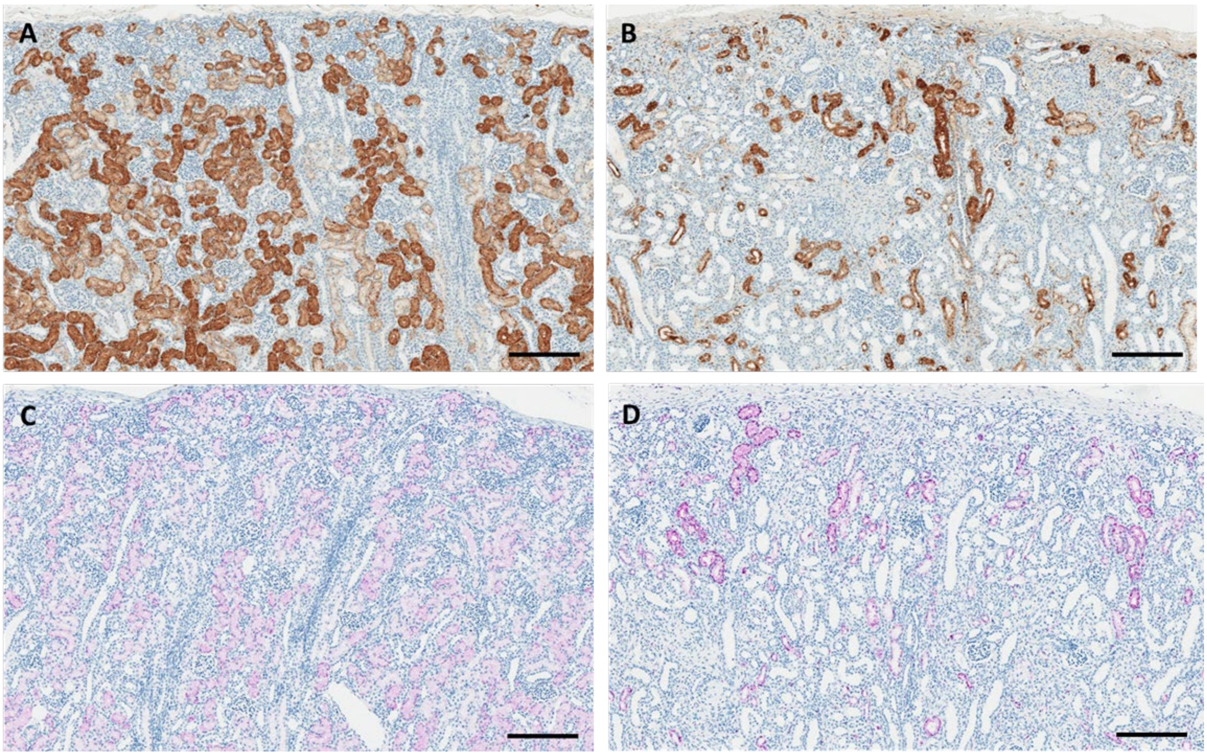

Figure S2. Immunohistochemistry and in situ hybridization. (A) Kidney, PND 30; Immunohistochemistry for RTR5001. Accumulation of RTR5001 into the proximal tubular cells (brown staining). (B) Kidney, PND 51, accumulation of RTR5001 in proximal tubular cells, fewer tubules and irregularly stained renal tubuli (brown staining). (C) Kidney, PND 51; in situ hybridization for RTR5001. Accumulation of RTR5001 into the proximal tubular cells (purple staining). (D) Kidney, PND 51, accumulation of RTR5001 in proximal tubular cells, fewer tubules and irregularly stained renal tubuli (purple staining). (Scale bar: A, B, C, and D = 200 μM).

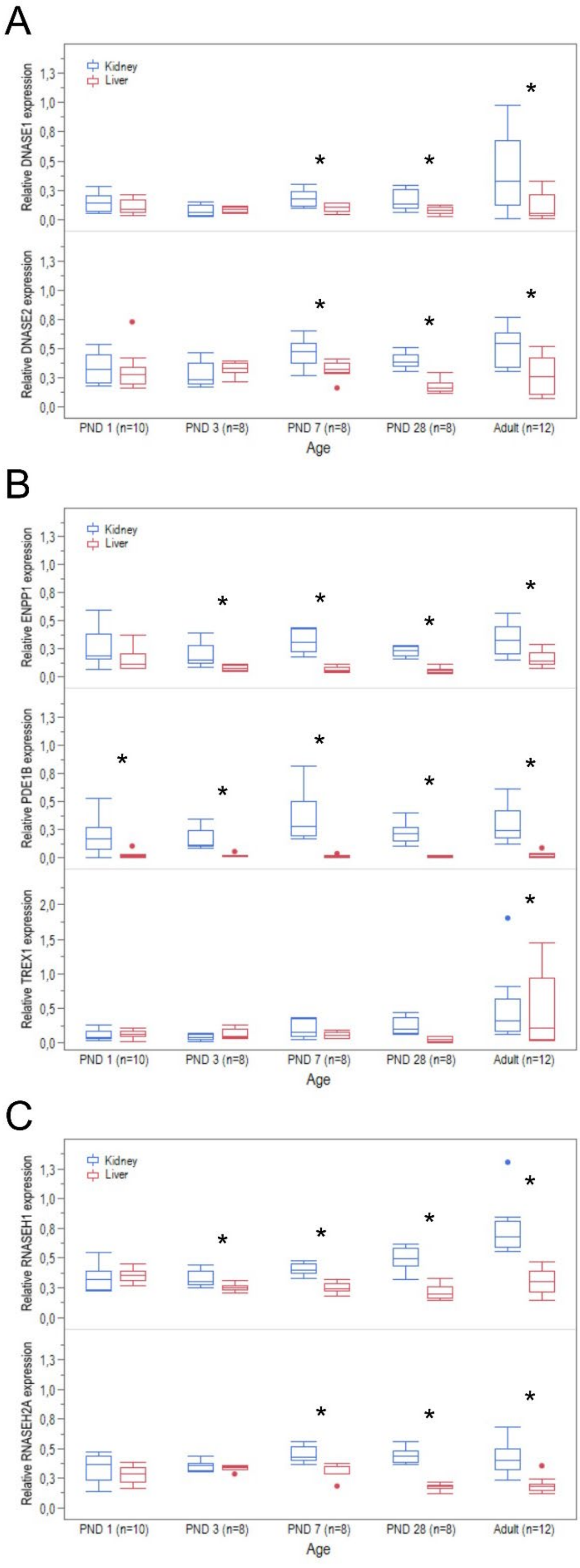

Figure S3. Relative gene expression of nucleases for ASO metabolism and pharmacologic activity between kidney and liver over time in post-natal and adult Göttingen Minipig. Endonucleases: *DNASE1*, and *DNASE2* (A); 3'-exonucleases: *ENPP1*, *PDE1B*, and *TREX1* (B); and *RNASEH1* and *RNASEH2A* (C) expression difference between the kidney and liver was considered significant (*) if $p < 0.05$.

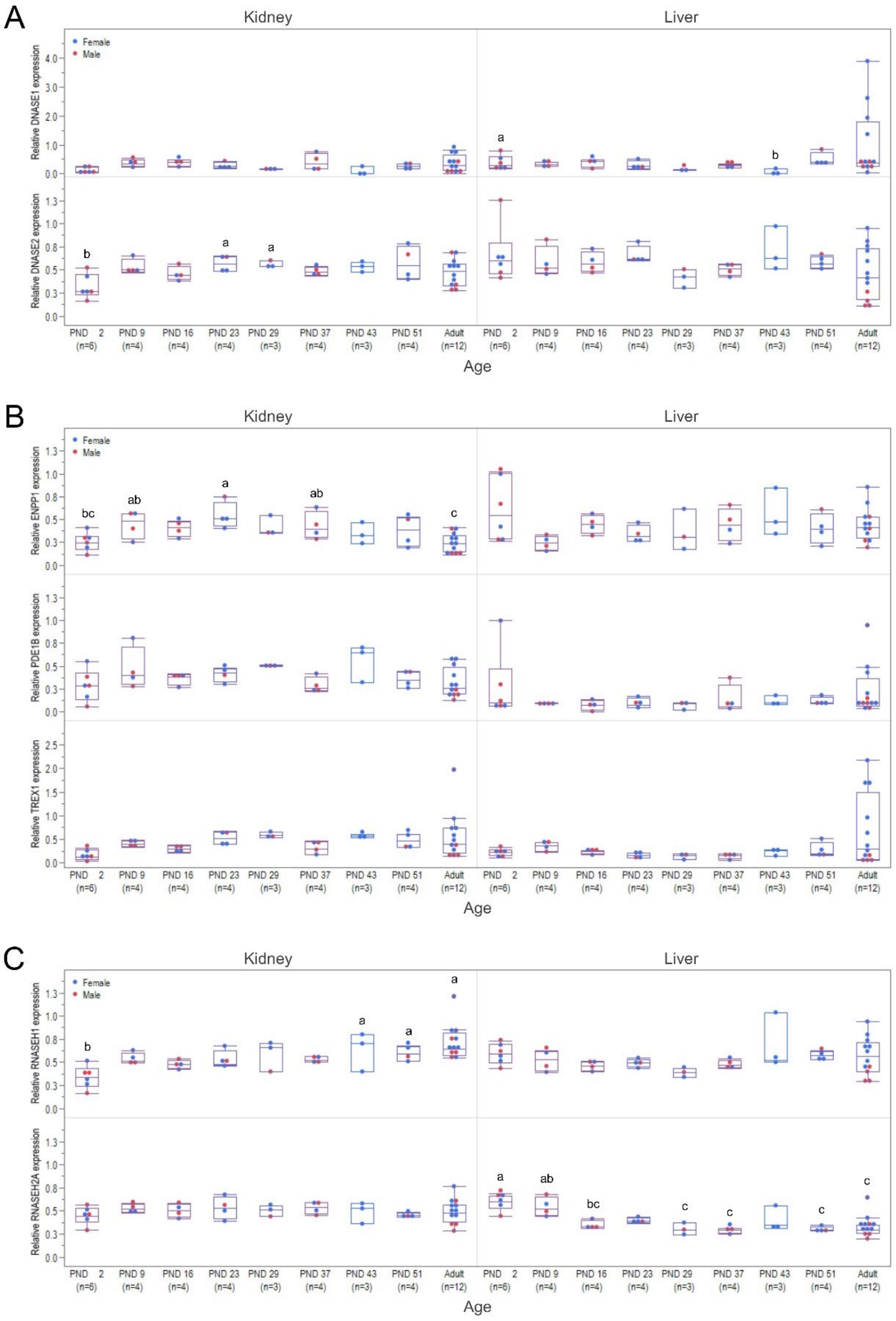

Figure S4. Relative gene expression of nucleases for ASO metabolism and pharmacologic activity over time in the developing and adult Göttingen Minipig samples from the investigational toxicity study. Endonucleases: *DNASE1*, and *DNASE2* (A); 3′-exonucleases: *ENPP1*, *PDE1B*, and *TREX1* (B); and *RNASEH1* and *RNASEH2A* (C) were evaluated in the kidney and liver samples from the investigational toxicity study. Different letters indicate significantly different expression in the age groups. Gene expression difference was considered significant if $p < 0.05$. Statistical analysis on liver *DNASE2*, *TREX1*, and *RNASEH1*, and kidney *TREX1* was not performed as sex-age interaction was detected on previous analysis of the biobank samples.

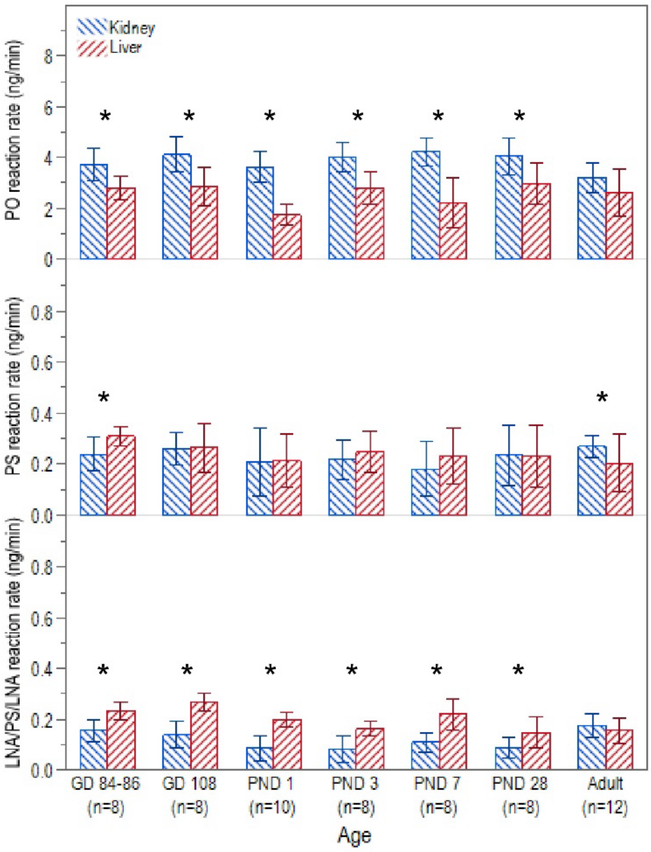

Figure S5. Estimated reaction rate towards the isosequential ASOs: unmodified (PO), all-PS (PS), and LNA/PS/LNA gapmer in the kidney and liver homogenates from the developing and adult Göttingen Minipig. Difference between the kidney and liver was considered significant (*) if $p < 0.05$.

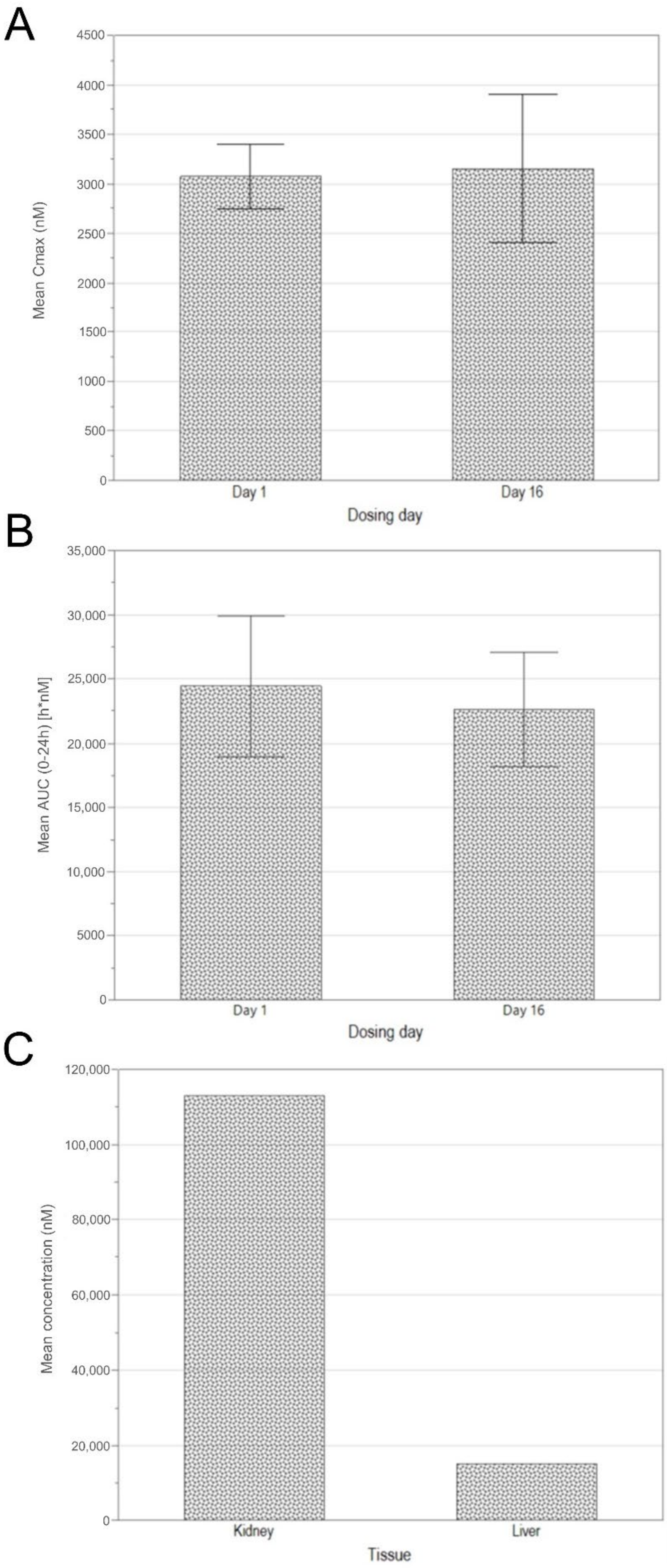

Figure S6. Published mean ± SD exposure levels in the plasma (A); mean ± SD maximum plasma concentration (Cmax) (B); and mean exposure levels in the kidney and liver (C) data of adult Göttingen Minipig after RTR5001 administration [1]. Cmax: Maximum concentration; AUC: Area under the curve.

Reference:

1. Braendli-Baiocco, A.; Festag, M.; Erichsen, K.D.; Persson, R.; Mihatsch, M.J.; Fisker, N.; Funk, J.; Mohr, S.; Constien, R.; Ploix, C.; et al. The minipig is a suitable non-rodent model in the safety assessment of single stranded oligonucleotides. *Toxicol. Sci.* **2017**, *157*, 112–128, doi:10.1093/toxsci/kfx025.
